# The Isolation and Characterization of ACE Inhibitory Peptides from *Pelodiscus sinensis* Wiegmann Meat and Their Effects on the Viability of HSC-T6 Cells

**DOI:** 10.3390/foods15162805

**Published:** 2026-08-11

**Authors:** Fangze Jiao, Xueqin Sun, Tianfeng Zhang, Xun Li, Guanglan Zheng, Lini Que, Yaming Liang, Pengying Liao

**Affiliations:** College of Pharmacy, Guangxi University of Chinese Medicine, Nanning 530200, China; jiaofangze2024@stu.gxtcmu.edu.cn (F.J.); sun_yue1998@163.com (X.S.); zhangtianfeng2023@stu.gxtcmu.edu.cn (T.Z.); lixun2024@stu.gxtcmu.edu.cn (X.L.); zgl2875161173@outlook.com (G.Z.); qln18176924139@outlook.com (L.Q.); 18177508352@163.com (Y.L.)

**Keywords:** ACE inhibitory peptides, turtle meat, HPLC-MS/MS, molecular docking

## Abstract

This study aimed to isolate and identify angiotensin I-converting enzyme (ACE) inhibitory peptides from Chinese soft-shelled turtle (*Pelodiscus sinensis* Wiegmann) meat. A total of 80 peptides were identified from the active fractions of the papain hydrolysate of the *Pelodiscus sinensis* meat water-soluble protein, including 78 unique peptides; among them, 47 had identification scores above 50. Of these, 10 peptides were predicted to possess ACE inhibitory potential, good water solubility, and no toxicity and were therefore synthesized for further evaluation. Peptide YK-17 showed the strongest ACE inhibitory activity among the synthesized peptides, with an IC_50_ value of 479.3 μM, while peptide GK-18 possessed the strongest inhibitory effect on the viability of the hepatic stellate cell line HSC-T6. Molecular docking further indicated that YK-17 formed more hydrogen bonds with N-ACE than with C-ACE, suggesting that YK-17 exhibited stronger hydrogen bonding interactions with N-ACE. These findings suggest that *Pelodiscus sinensis* meat is a promising source of functional peptides with potential antihypertensive activity and liver fibrosis-related bioactivity.

## 1. Introduction

The Chinese soft-shelled turtle (*Pelodiscus sinensis* Wiegmann), also known as turtle, belongs to the family Trionychidae [1]. It is an important freshwater aquatic species in Zhejiang, Jiangsu, Hubei, Guangxi and other places in China [2], with high medicinal and food value. The earliest existing pharmacological monograph, *Shennong’s Herbal Classic*, contains turtle shell (*Carapax trionycis*) [3]. According to the Chinese Materia Medica, turtle meat is sweet and neutral in nature, belongs to the liver meridian, nourishes yin, tonifies the kidney, and clears away deficiency heat [4]. The meat is also rich in proteins, peptides, amino acids and other active substances [5], with anti-hepatic fibrosis, anti-tumor, immunomodulation and other biological activities [6,7]. Given that turtle meat is rich in protein and poor in fat [8], most active peptides in proteins are released by protease-catalyzed hydrolysis, exerting their good biological activities [9]. For example, the angiotensin I-converting enzyme (ACE) inhibitory activity of turtle meat was significantly enhanced after enzymatic hydrolysis compared with that before hydrolysis, indicating that enzymatic hydrolysis facilitated the release of bioactive peptides from native proteins. This is consistent with previous studies showing that soft-shelled turtle-derived protein hydrolysates and peptides possess diverse biological activities, including ACE inhibitory, antioxidant, and anti-advanced glycation end product (AGE) formation effects [10,11,12,13]. In particular, previous studies have identified novel ACE inhibitory peptides from soft-shelled turtle meat hydrolysates; for example, a specific protease-assisted hydrolysis and screening workflow led to the identification of the peptide IEWEF with confirmed in vitro ACE inhibitory activity. Together, these findings demonstrate the potential of turtle meat proteins as a source of functional peptides [11]. Beyond their biofunctional potential, turtle meat proteins also represent a promising resource for high-value utilization through deep processing. The discovery and development of bioactive peptides from turtle meat may not only create new value-added products but also enhance the economic output of aquatic resources and broaden their application in functional foods, nutraceuticals, and related health products [14]. This value-added potential further supports the investigation of turtle meat-derived peptides as functional ingredients.

ACE is a key Renin–angiotensin system (RAS) enzyme, widely distributed in many human tissues and organs [15]. Its function in the classical metabolic pathway of the RAS is to maintain the stability of blood pressure. At the same time, the local RAS is associated with organ fibrotic lesions [16]. Clinically used ACE inhibitors, such as captopril, lisinopril, and enalapril, show blood pressure-lowering function and also exert certain effects on the main components of the local tissue RAS, including an inhibitory effect on organ fibrotic lesions [17,18,19]. However, the long-term use of these drugs induces adverse reactions, such as dry cough, rash, and tachycardia. Nonetheless, ACE inhibitory peptides in natural proteins are safe and effective, with a few side effects [20]. For example, an ACE inhibitory peptide, IVGFPAYGH, identified from the in vitro digestion of pork sausage with the partial substitution of NaCl by KCl, has been reported to protect against high-sodium diet-induced liver injury in mice, potentially through the suppression of hepatic RAS activation; notably, this peptide has also been shown to increase ACE2 and NO levels and decrease ACE, Ang II, and TGF-β1 levels in Ang I-treated HUVECs [21].

Hepatic stellate cells (HSC) are the main fibroblasts in the liver [22]. They usually rest in normal livers and are activated and transformed into mesenchymal myofibroblasts after liver injury. HSC produces extracellular matrix (ECM) components of fibrotic tissues and a wide range of pro-inflammatory cytokines and chemokines [22,23]. The RAS consists of angiotensin II (Ang II), with ACE and AT_1_R forming the classical ACE-Ang II-AT_1_R pathway in the RAS, which could promote hepatic fibrosis formation [24]. Through this pathway, ACE is closely associated with the regulation of HSC behavior. The main function of ACE is to excise the Ang I carboxy-terminal dipeptide to generate Ang II, which regulates the contraction, proliferation, and activation of HSCs and the inhibition of their apoptosis [25]. Therefore, ACE may indirectly promote liver fibrosis through the Ang II-mediated facilitation of HSC proliferation and activation. Ohishi et al. [17] investigated the therapeutic effect of lisinopril on carbon tetrachloride-induced hepatic fibrosis in rats and revealed that lisinopril could significantly inhibit liver fibrosis in rats. Therefore, inhibiting the activity of ACE and its related factors in the RAS may positively affect the treatment of liver fibrosis. HSC activation and proliferation are important events in hepatic fibrogenesis. Therefore, the inhibition of HSC-T6 cell viability may serve as a preliminary in vitro indicator of potential antifibrotic activity.

In the human body, two forms of ACE are prevalent in all tissues, with two active centers, the N-terminal structural domain and the C-terminal structural domain [26]. These two structural domains have different physiological functions. The C-terminal structural domain, closer to the cell membrane surface, regulates blood pressure and cardiovascular function. Its main function is to regulate arterial blood pressure and electrolyte homeostasis through the RAS and kinin-releasing enzyme kinin systems. On the other hand, the N-terminal structural domain is closely associated with the diseases such as hepatic fibrosis. The variability in the affinity of ACE inhibitors in the N/C structural domains influences their physiological functions.

Based on the structural and functional differences between the N- and C-terminal domains of ACE, it is reasonable to propose that soft-shelled turtle meat may contain ACE inhibitory peptides with distinct affinities for these two domains. Considering the close relationship between the N-terminal domain and hepatic fibrosis, peptides with greater affinity for the N-terminal domain may exhibit a stronger inhibitory effect on HSC-T6 cell viability, thereby indicating their potential antifibrotic properties.

Although previous studies have confirmed that the papain enzymatic digest of turtle meat has strong ACE inhibitory activity [21], there are obvious research gaps: First, the specific composition of ACE inhibitory peptides in the papain enzymatic digest of turtle meat has not been clearly identified, and the correlation between peptide composition and ACE inhibitory activity remains unclear. Second, the interaction mechanism between these turtle-derived ACE inhibitory peptides and the N/C-terminal domains of ACE has not been explored, especially the difference in binding affinity between peptides and the two structural domains. Third, the in vitro ACE inhibitory activity of the screened peptides and their regulatory effect on HSC-T6 cell viability (a key cell in hepatic fibrosis) have not been systematically evaluated, which limits the in-depth development and application of the medicinal value of turtle meat.

This study aimed to clarify the composition and bioactivity of ACE inhibitory peptides from the papain enzymatic digest of turtle meat and their interaction mechanism with ACE N/C-terminal domains. The papain enzymatic digest from turtle meat was isolated and purified, peptides were identified, and their activity was also predicted. In addition, ACE inhibitory peptides were screened out, and their ACE inhibitory activity was evaluated in vitro, as well as the viability of HSC-T6 cells. Molecular docking software was also used to evaluate the differences in the interaction between peptides and the ACE N/C-terminal structural domains. The findings obtained from this study will help to strengthen in-depth research about the medicinal value of turtle meat.

## 2. Materials and Methods

### 2.1. Materials and Reagents

*Pelodiscus sinensis* meat protein hydrolysate (PMPH) was prepared in the laboratory. ACE was derived from rabbit lungs. Hippuryl–Histidyl–Leucine (HHL) was sourced from Sigma-Aldrich Co., St. Louis, MO, USA. Methanol and acetonitrile were purchased from Thermo Fisher Scientific, Waltham, MA, USA, and are chromatography-grade. Sephadex G-25 was obtained from Beijing Solarbio Science & Technology Co., Ltd., Beijing, China. The C18 column (150 mm × 4.6 mm, 5 μm) was sourced from Thermo Fisher Scientific, Waltham, MA, USA, and YMC-Pack ODS-A column (250 mm × 4.6 mm, 5 μm) from YMC Corporation, Kyoto, Japan. The rat hepatic stellate HSC-T6 cells (CL-0116), HSC-T6 cell-specific medium, high-glucose Dulbecco’s Modified Eagle Medium (DMEM), and fetal bovine serum (FBS) were procured from Wuhan Pricella Biotechnology Co., Ltd., Wuhan, China. The HSC-T6 cell line (RRID: CVCL_0315) was originally established and characterized by Vogel [27]. Additionally, 0.25% EDTA–trypsin, the penicillin–streptomycin mixture (100×), and phosphate-buffered solution (PBS) were sourced from Beijing Solarbio Science & Technology Co., Ltd., Beijing, China. Cell Counting Kit-8 (CCK-8) was sourced from Shanghai Baisai Biotechnology Co., Shanghai, China. The peptides (purity > 95%) were synthesized by Shanghai Gill Biological Co., Shanghai, China.

*Pelodiscus sinensis* meat protein hydrolysate (PMPH) was prepared from freeze-dried turtle meat mince. The sample was mixed with distilled water at a material-to-liquid ratio of 1:10 (*w*/*v*) and extracted under stirring at room temperature for 4 h. The extract was then centrifuged at 2000× *g* for 20 min at room temperature, and the supernatant was collected. This extraction procedure was repeated three times, and the collected supernatants were combined and freeze-dried to obtain the water-soluble turtle meat protein, which was stored at −20 °C until use. The water-soluble protein was subsequently hydrolyzed with papain. Based on single-factor experiments and response surface methodology optimization, the hydrolysis conditions were set as follows: material-to-liquid ratio 1:40 (*w*/*v*), enzyme dosage 8653 U/g, hydrolysis temperature 38 °C, pH 6.9, and hydrolysis time 4 h. The optimized process was verified in triplicate, and the degree of hydrolysis of the resulting hydrolysate was 64.94 ± 0.17%. The obtained hydrolysate was designated as PMPH and used for subsequent experiments.

### 2.2. Main Instruments and Equipment

The main instruments used in this study included a BSA224S electronic balance (Sartorius Scientific Instrument (Beijing) Co., Ltd., Beijing, China); a DBS-100 automatic sample collector (Shanghai Qingpu Huxi Instrument Factory Co., Ltd., Shanghai, China); a Waters 2695 high-performance liquid chromatograph equipped with an Empower data processing system (Waters Corporation, Milford, MA, USA); an LC3000N semi-preparative high-performance liquid chromatograph (Beijing Innovative Tong Heng Chromatography Technology Co., Ltd., Beijing, China); a UV-1780 ultraviolet spectrophotometer (Shimadzu Instruments (Suzhou) Co., Ltd., Suzhou, China); a Q-Exactive high-resolution mass spectrometer with an electrospray ionization (ESI) source (Thermo Fisher Scientific, Waltham, MA, USA); an MCO-18AIC CO_2_ constant-temperature incubator (PHCbi Corporation, Tokyo, Japan); a CKX41 inverted microscope (Olympus Corporation, Tokyo, Japan); an Infinite 200Pro microplate reader (Tecan Group Ltd., Männedorf, Switzerland); and an ST16 centrifuge (Thermo Fisher Scientific, Waltham, MA, USA).

### 2.3. Sephadex G-25 Separation

The Sephadex G-25 [28] gel was soaked overnight in ultrapure water to ensure full swelling, then loaded into the glass chromatography column at once to avoid air bubbles or faults in the gel column. Next, 2 to 3 column volumes were equilibrated using ultrapure water. Subsequently, lyophilized PMPH was dissolved in ultrapure water (30 mg/mL), filtered through a 0.45 μm microporous filter membrane to remove impurities, and separated by Sephadex G-25 (Φ 1.6 × 110 cm).

Precisely, 0.5 mL of sample volume was separated at a time. The flow rate was adjusted to 0.3 mL/min with ultrapure water as the eluent under isocratic elution conditions. The stream fractions were collected with an automatic sample collector (Shanghai Qingpu Huxi Instrument Factory Co., Ltd., Shanghai, China) at 10 mL per tube, and a total of 120 tubes were collected. The absorbance of each stream fraction was measured at 220 nm using an ultraviolet spectrophotometer (Shimadzu Instruments (Suzhou) Co., Ltd., Suzhou, China). In addition, the elution curve was plotted with the sample bottle number as the *X*-axis and the absorbance of the sample as the *Y*-axis. Next, the fractions were combined and freeze-dried, and the ACE inhibitory activity of each fraction was determined. Finally, the fraction with the highest ACE inhibitory activity was selected for further purification.

### 2.4. Semi-Preparative RP-HPLC Purification

The active components obtained from Sephadex G-25 gel separation were dissolved in ultrapure water to form a 10 mg/mL sample solution. The obtained solution was filtered through a 0.45 μm microporous membrane, and 100 μL of the filtrate was purified by semi-preparative RP-HPLC [29] using a YMC-Pack ODS-A (250 × 10 mm, 5 μm) chromatographic column. The chromatographic conditions were as follows: we used a flow rate of 3.0 mL/min and detection wavelength at 220 nm, mobile phase A was acetonitrile, and mobile phase B was ultrapure water, for 0–15 min, with 5% A isocratic elution. The eluate was monitored at 220 nm, and fractions corresponding to each chromatographic peak were collected separately. The samples were injected repeatedly to enrich each collected fraction. Finally, after lyophilization by vacuum freeze drying (Martin Christ Gefriertrocknungsanlagen GmbH, Osterode am Harz, Germany), ACE inhibitory activity was determined to identify the most bioactive fraction.

### 2.5. Peptide Identification

#### 2.5.1. HPLC-MS/MS Data Acquisition

Peptides in samples were identified using a nanoscale flow rate HPLC liquid system (EASY-nLC) in tandem with a Q Exactive mass spectrometer (Thermo Fisher Scientific, Waltham, MA, USA) [28]. The chromatographic column used was a Thermo Scientific C18 Easy-Column (100 μm × 2 cm), followed by a C18-reversed phase analytical column (Thermo Scientific Easy Column, 10 cm long, 75 μm inner diameter, 3 μm resin) for separation. The mobile phases consisted of 0.1% formic acid aqueous solution and 84% acetonitrile aqueous solution (containing 0.1% formic acid) at a 300 nL/min flow rate. The injection volume was 40 μL, the sample loading amount was 20 μg, the column temperature was maintained at room temperature (20 °C), and the total run time was 65 min.

A Q-Exactive high-resolution mass spectrometer (Thermo Fisher Scientific, Waltham, MA, USA) was used for mass spectrometry analysis. The mass spectrometry conditions were: ion source—electrospray ionization source; positive ion detection modes—Full Scan (*m*/*z* 300–1800) and data-dependent secondary mass spectrometry (TopN = 10) with resolutions of 70,000 (primary mass spectrometry) and 17,500 (secondary mass spectrometry), respectively; collision mode—high-energy collisional dissociation with a collision energy of 30 eV. This experiment was qualitative and aimed to characterize the peptide composition of the fractions; therefore, no replicate analysis was performed.

#### 2.5.2. Database Search and Peptide Identification

The raw data from the mass spectrometry analysis were processed using MaxQuant software (version 1.6.14.0) [30] for peptide sequence identification. Subsequently, the obtained peptides were searched in the protein database uniprot_Pelodiscus_sinensis_20952_20220725. The search criteria were fixed modification Carbamidomethyl (C), variable modification Oxidation (M), Acetyl (Protein N-term), and a false discovery rate (FDR) ≤ 1%. Peptides with an identification score higher than 50 were accepted for further analysis [31].

### 2.6. Bioinformatic Analysis of Peptides

The BIOPEP-UWM database (http://www.uwm.edu.pl/biochemia/index.php/pl/biopep, accessed on 10 August 2022) [32] was used to identify the reported and unreported peptides. Peptide toxicity was predicted using the ToxinPred (https://webs.iiitd.edu.in/raghava/toxinpred/, accessed on 10 August 2022) online website. In addition, peptide water solubility was predicted using a proteomics tool (http://www.innovagen.com/, accessed on 10 August 2022). Finally, the potential biological activity of the peptides was predicted by the Peptide Ranker database (https://peptide.ucd.ie/peptideranker/, accessed on 10 August 2022) [11].

### 2.7. In Vitro ACE Inhibitory Activity of Peptide Fragments

As previously described, a peptide ACE inhibitory activity assay was performed in vitro [33]. The samples were prepared to a certain concentration (1.0 mg/mL), and subsequently, a curve was fitted with Log (peptide concentration) against the ACE inhibition rate, and the corresponding sample concentration was calculated when the ACE inhibition rate was 50%, the IC_50_ value of ACE inhibition in the sample. For IC_50_ determination, the samples were tested at concentrations of 0.125, 0.25, 0.5, 1, and 2 mg/mL. All experiments were performed in triplicate.

### 2.8. Preliminary In Vitro Evaluation of Inhibitory Effect of Peptide Fragments on HSC-T6 Cell Viability

#### 2.8.1. Peptide Solution and Medium Preparation

A complete medium consisting of high-glucose DMEM supplemented with 10% FBS and a 1% penicillin–streptomycin mixture was stored at 4 °C away from light. The cell freezing solution consisted of a DMEM containing 40% FBS and 50% dimethyl sulfoxide (DMSO) [34]. For peptide sample preparation, DMEM containing 10% FBS was added to peptide samples (100 μM) to dissolve them (DMSO final concentration should not exceed 0.5%). A peptide solution with a concentration of 100 μM was prepared and then filtered through a 0.22 μm sterile filter head.

#### 2.8.2. Effect of Peptides on Viability of HSC-T6 Cells

Three treatment groups were prepared, including a blank group, a control group, and a peptide group. Experiments were performed using 96-well plates, and 100 μL of complete medium was added in the blank group, 100 μL of cell suspension with a cell density of 5 × 10^4^ cells/mL was added in the control group, and 100 μL of complete medium containing a peptide concentration of 100 μM was added in the peptide group.

The HSC-T6 cells in the logarithmic growth phase were adjusted to a cell density of 5 × 10^4^ cells/mL; inoculated in a 96-well plate, with 100 μL of cell suspension/well; and incubated at 37 °C and 5% CO_2_ for 24 h. After adherent cell growth to the wall, the old culture medium was aspirated off, and 100 μL peptide-containing medium was added to every group with 4–6 replicate wells per treatment. The wells were incubated for 24 h or 48 h, and the drug-containing medium was aspirated off before adding 100 μL complete culture medium, followed by 10 μL CCK-8 [35]. Next, the culture plates were incubated at 37 °C and 5% CO_2_ for 2 h, before measuring the OD value at the 450 nm wavelength. Finally, the cell viability of HSC-T6 cells was calculated according to the following formula.

(1) $$Cell\;viability\;(\%)=\frac{{OD}_{drug}-{OD}_{blank}}{{OD}_{control}-{OD}_{blank}}\times 100\%$$

### 2.9. Molecular Docking

The protein structures of two different structural domains of angiotensin-converting enzyme, N-ACE (PDBID 2C6N) and C-ACE (PDBID 1O8A), were downloaded from the RCSB Protein Data Bank (http://www.rcsb.org/pdb/home/home.do, accessed on 3 November 2022) [36]. The two protein files were opened separately with PyMOL software (version 3.0) to remove water molecules and add hydrogen atoms. The 2D structures of the peptides were generated with ChemDraw software (version 14.0) and then converted into 3D structures by ChemDraw 3D software (version 14.0).

The ligands and proteins were separately opened in Auto Dock Tools. For N-ACE, the docking box center was set at x = −23.262, y = −19.269, and z = −37.892, with dimensions of 60 Å × 60 Å × 60 Å. For C-ACE, the docking box center was set at x = 40.562, y = 37.388, and z = 43.476, with dimensions of 60 Å × 60 Å × 60 Å [37]. For the accuracy of the calculation results in the docking module, the modes were set to 10 and the exhaustiveness to 16, with default values for all other parameters.

### 2.10. In Silico Analysis of Gastrointestinal Digestion and Stability of Peptides

An in silico gastrointestinal digestion strategy, incorporating enzymatic cleavage with pepsin trypsin and chymotrypsin, was employed to evaluate the stability of the target peptides [38]. The BIOPEP-UWM database (https://biochemia.uwm.edu.pl/biopep/start_biopep.php, accessed on 18 July 2026) [32] was used to predict the theoretical peptide fragments released from the target peptides after simulated gastrointestinal digestion through in silico hydrolysis. The “enzyme action” program in BIOPEP-UWM was applied, and pepsin (pH 1.3, EC 3.4.23.1), trypsin (EC 3.4.21.4), and chymotrypsin (EC 3.4.21.1) were selected to simulate the gastrointestinal digestion of the peptide sequences. Moreover, following in silico digestion, the generated fragments were analyzed in the BIOPEP-UWM database to identify their potential biological activities.

### 2.11. Statistical Analysis of Data

All data were processed using Excel 2019 and analyzed using GraphPad Prism 5.0 software. All experiments were performed with three biological replicates and three technical replicates. Statistical differences among groups were evaluated by a one-way analysis of variance (ANOVA), followed by Bonferroni’s post hoc test where appropriate. A *p* value < 0.05 was considered statistically significant.

## 3. Results and Discussion

### 3.1. Isolation and Purification of ACE Inhibitory Active Components

#### 3.1.1. Separation of PMPH by Sephadex G-25

**Figure 1:**
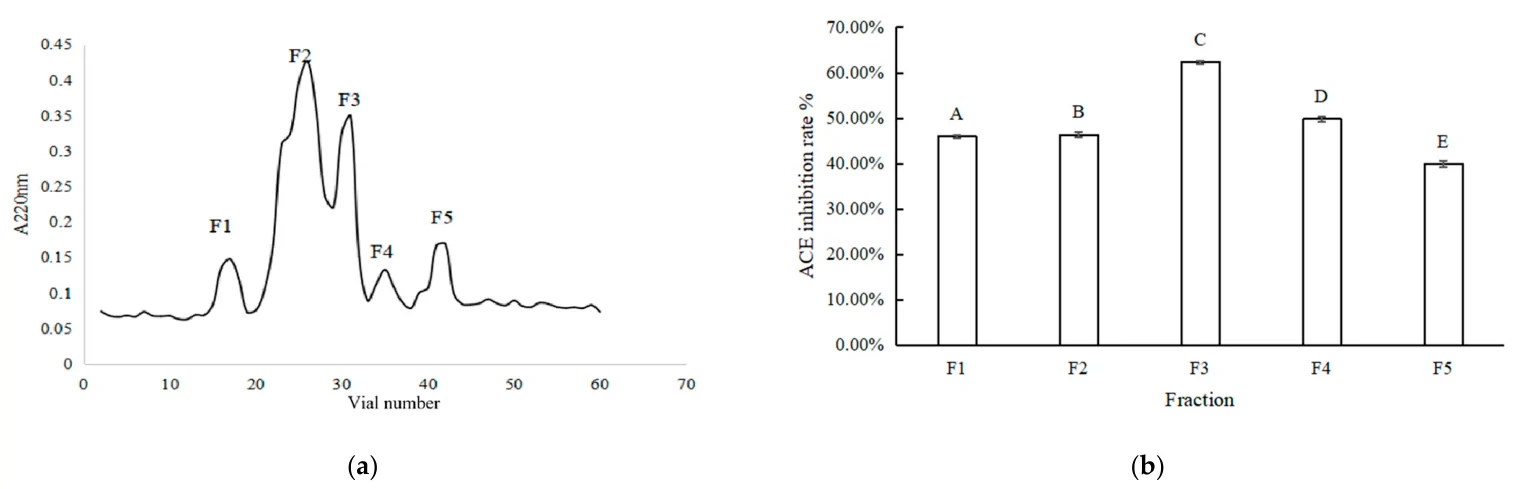
(**a**) Chromatogram of Sephadex G-25 of PMPH. (**b**) ACE inhibitory activity of fractions obtained from PMPH by Sephadex G-25 gel filtration chromatography. Different letters represent statistically significant difference (*n* = 3, *p* < 0.0001).

The elution curve of PMPH separated on Sephadex G-25 is shown in Figure 1a. PMPH was divided into five fractions (F1 to F5), and their ACE inhibitory activity is shown in Figure 1b. As shown in Figure 1b, the ACE inhibitory activities of the five fractions were significantly different. The activity of F5 is relatively low, while F1, F2 and F4 have moderate activity, which may be related to the types and contents of peptides contained in different fractions. Fraction F3 exhibited the strongest ACE inhibitory activity (62.35 ± 0.35%) at 2.0 mg/mL, which was significantly higher than that of the other fractions (*p* < 0.05).

Gel filtration chromatography separates components based on molecular weight, with higher-molecular-weight fractions eluting earlier and lower-molecular-weight fractions eluting later. Fraction F3 was located within the medium-molecular-weight range. Although many studies have reported that most ACE inhibitory peptides have molecular weights below 3 kDa, the lowest-molecular-weight fraction in the present study showed relatively weak ACE inhibitory activity. This result suggests that molecular weight alone is not the determining factor for ACE inhibitory activity. A possible explanation is that the low-molecular-weight fraction contained substantial amounts of free amino acids and inactive peptide fragments, which may have reduced its overall activity. In contrast, ACE inhibitory activity is more likely governed by structural features such as peptide amino acid sequence, side chain hydrophobicity, and the compatibility of the peptide with the ACE active sites. Similar observations have also been reported in previous studies, in which the fractions with the strongest ACE inhibitory activity were not necessarily the lowest-molecular-weight fractions [39]. Therefore, the stronger activity of F3 may be attributed to the presence of peptide components with more favorable interactions with ACE binding sites.

#### 3.1.2. Semi-Preparative RP-HPLC Separation of Fraction F3

**Figure 2:**
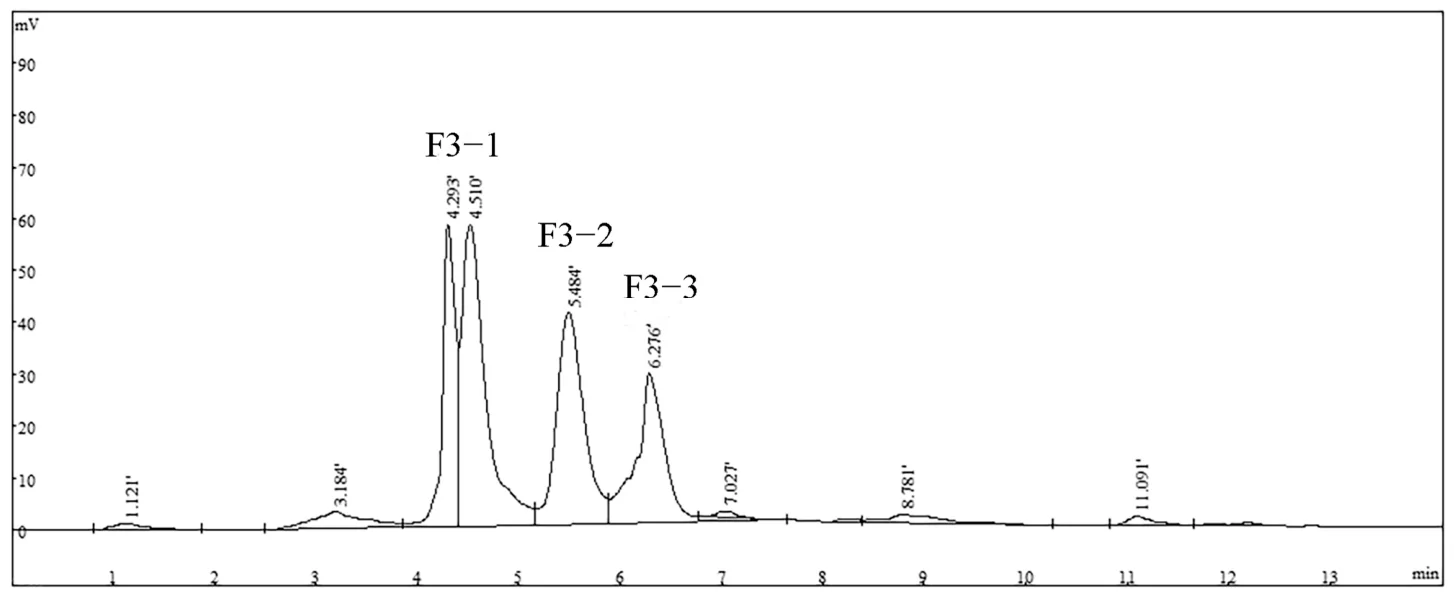
Chromatogram of fraction F3 purified by semi-preparative method.

**Figure 3:**
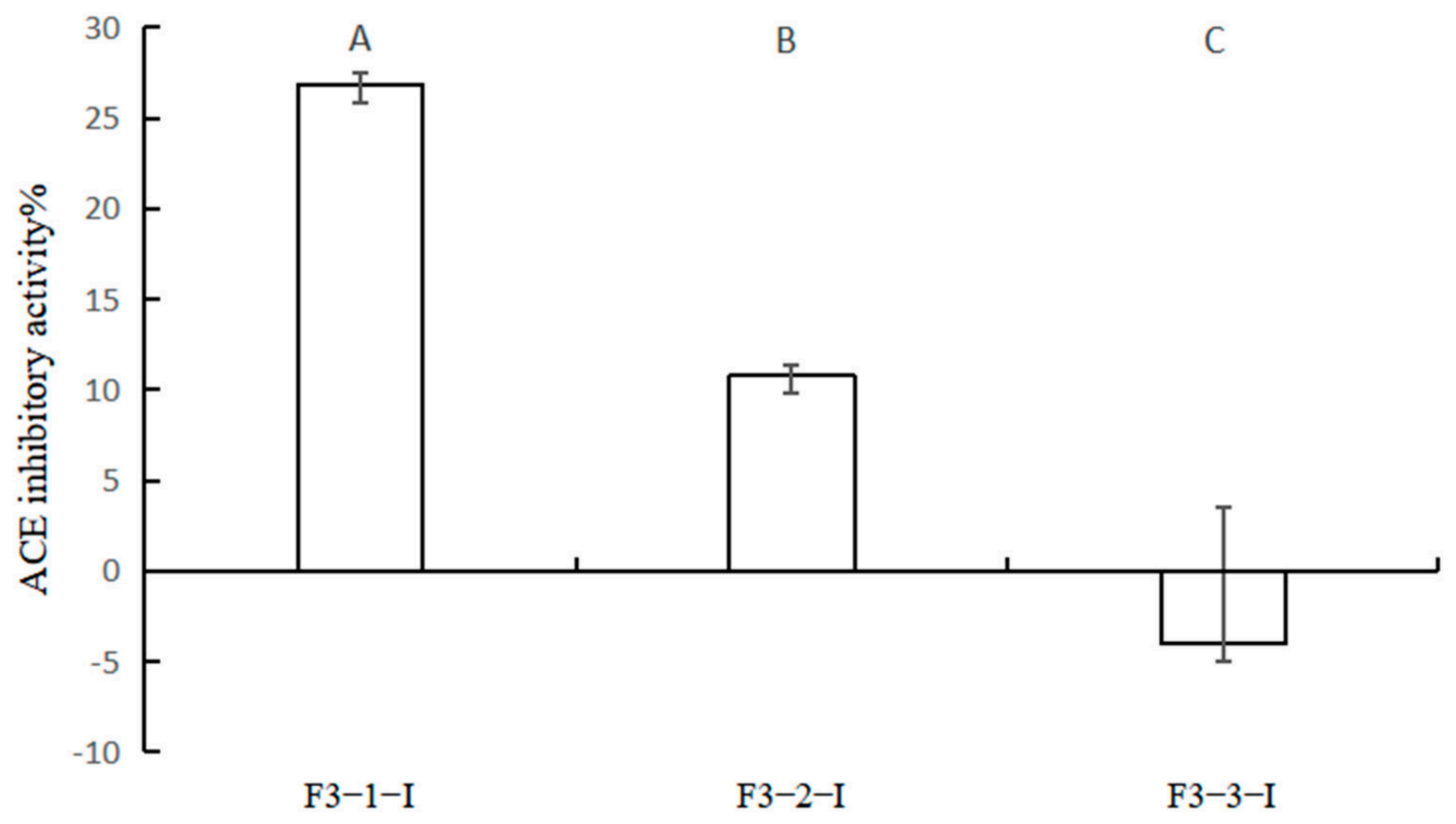
ACE inhibitory activity of each component of semi-preparative RP-HPLC-purified F3. Different letters represent statistically significant difference (*n* = 3, *p* < 0.05).

The separation of fraction F3 by semi-preparative RP-HPLC yielded three components, F3-1, F3-2, and F3-3, whose chromatograms are shown in Figure 2. The continuous purification of components F3-1, F3-2 and F3-3 by semi-preparative RP-HPLC yielded components F3-1-I, F3-2-I and F3-3-I. The purpose of the further purification of F3-1, F3-2 and F3-3 is to remove impurities that may be contained in these components so as to obtain purer peptide components, which is conducive to the accurate determination of their ACE inhibitory activity and subsequent peptide sequence identification. Their ACE inhibitory activity in vitro is shown in Figure 3. Based on the in vitro ACE inhibitory activity of components F3-1-I and F3-2-I, these two components were selected for peptide sequence identification.

This two-step separation and purification process is consistent with the semi-preparative RP-HPLC separation strategy of tilapia skin hydrolysate peptide segments [40], both of which the achieve effective separation of target components through optimized gradient elution. RP-HPLC separates peptides based on differences in hydrophobicity. Peptides with lower hydrophobicity elute earlier because of their weaker interaction with the stationary phase, whereas more hydrophobic peptides elute later. In this study, F3-1 eluted first, followed by F3-2 and F3-3. Interestingly, the purified component F3-1-I showed the strongest ACE inhibitory activity, F3-2-I showed the second highest activity, and F3-3-I exhibited no ACE inhibitory activity. This suggests that the subfractions with higher activity were not necessarily those with stronger hydrophobicity. A possible explanation is that peptides with better water solubility may more readily access the hydrophilic entrance region of ACE and interact more effectively with the active site, including Zn^2+^, through coordination and hydrogen bonding interactions, thereby contributing to stronger inhibitory activity. However, because the amount of the initial F3 fraction was limited, the RP-HPLC-derived subfractions were insufficient for peptide content determination and other quantitative analyses. Therefore, a quantitative comparison of ACE inhibitory activity before and after RP-HPLC purification could not be performed, and the purification efficiency of RP-HPLC for enriching active components could not be fully evaluated.

### 3.2. Peptide Identification Results

**Figure 4:**
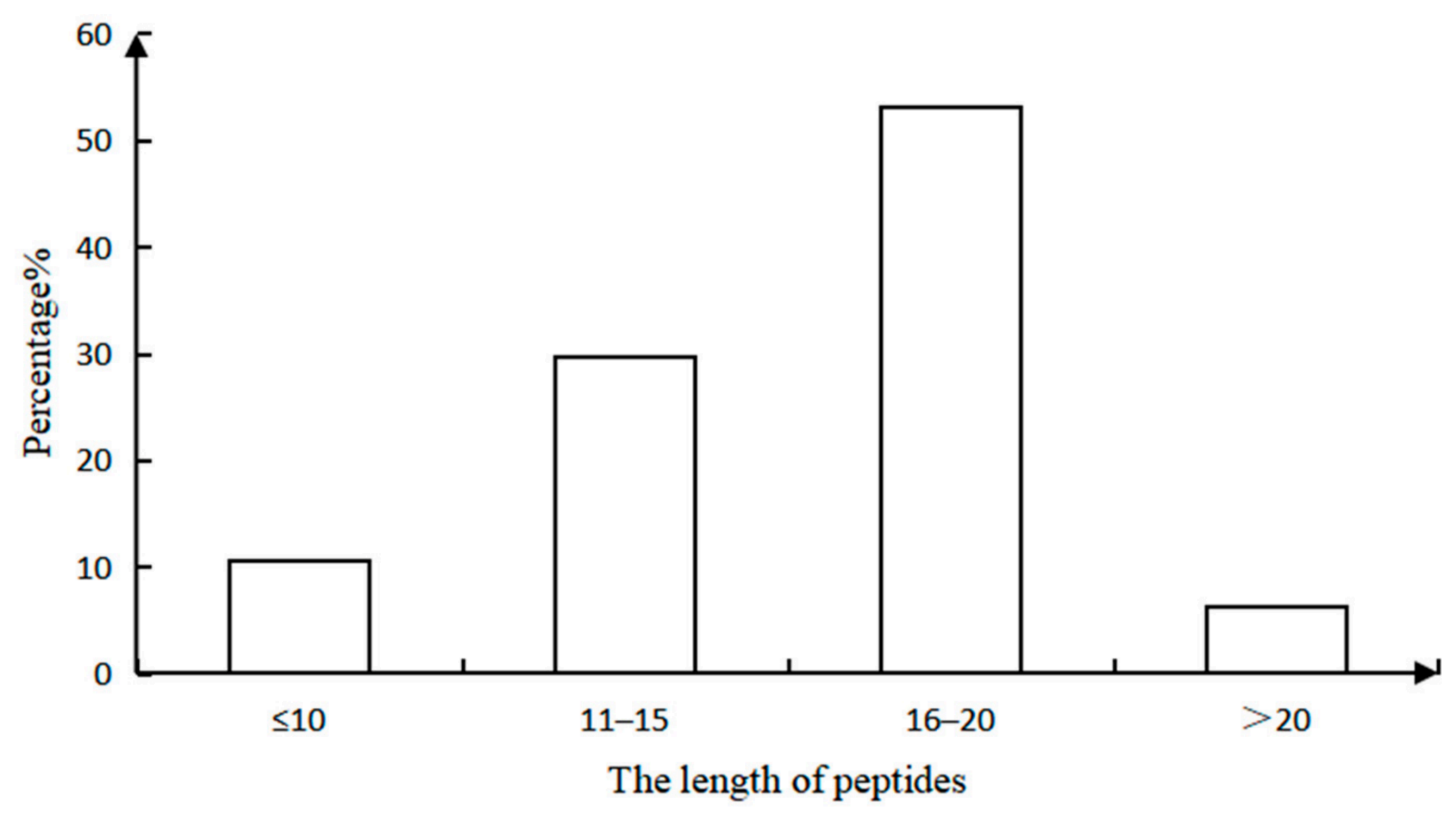
Peptide chain length distribution of turtle meat peptides.

**Figure 5:**
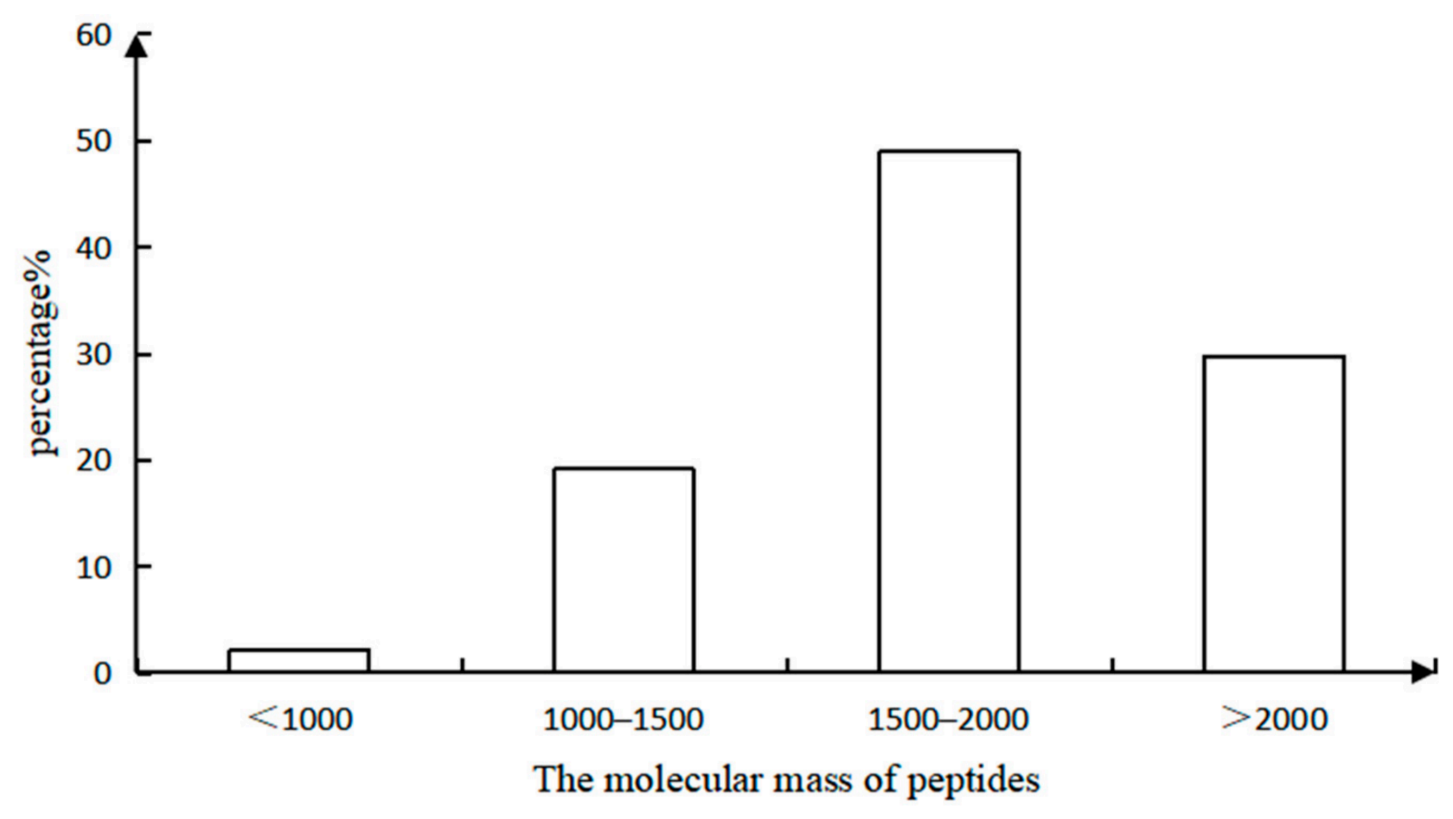
Molecular mass distribution of turtle meat peptides.

Eighty peptides were identified from the active fractions, including 78 unique peptides and 47 peptides with identification scores [41] above 50 (Table S1). In peptidomic analysis, a higher identification score generally indicates a more reliable peptide assignment. A score above 50 is generally considered to be a relatively reliable identification standard according to commonly used proteomic quality criteria and the relevant literature [31]. Therefore, the 47 peptides with scores above 50 were selected as the main research objects, which can reduce the error caused by inaccurate peptide identification and ensure the reliability of subsequent research. To better evaluate their potential ACE inhibitory activity, the 47 peptides were further analyzed for sequence length, molecular weight, and amino acid composition. The length and relative molecular mass distribution of these 47 peptides are shown in Figure 4 and Figure 5. Their lengths ranged from 9 to 23 amino acid residues, and their relative molecular masses were 1000–2600 Da. The length of most peptides was 16–20 amino acid residues, with a relative molecular mass distribution of 1500–2000 Da. This is noteworthy because an assessment of ACE inhibitory peptides of meat origin revealed that fractions with molecular weights < 3000 Da showed stronger ACE inhibitory activity than fractions with molecular weights > 3000 Da [42,43].

Previous studies [11] have shown that most ACE inhibitory peptides are relatively short, typically consisting of 2–12 amino acid residues with molecular masses below 1000 Da, which is generally considered favorable for access to and interaction with the ACE active site. In contrast, most peptides identified in the present study were composed of 16–20 amino acid residues and had molecular masses mainly distributed between 1500 and 2000 Da, suggesting that their relatively larger size may impose steric constraints on binding to the ACE active site. Nevertheless, these peptides may possess relatively good water solubility, which could facilitate their interaction with Zn^2+^ at the catalytic center of ACE and promote the formation of multiple hydrogen bonds with ACE.

The molecular weight distribution of the peptides from *Pelodiscus sinensis* meat identified in this study is consistent with that reported in a previous study [44]. The consistency with previous studies shows that the peptide identification results of this study are relatively reliable and also indicates that the molecular weight distribution of peptides obtained by the papain hydrolysis of *Pelodiscus sinensis* Wiegmann meat has certain regularity.

**Figure 6:**
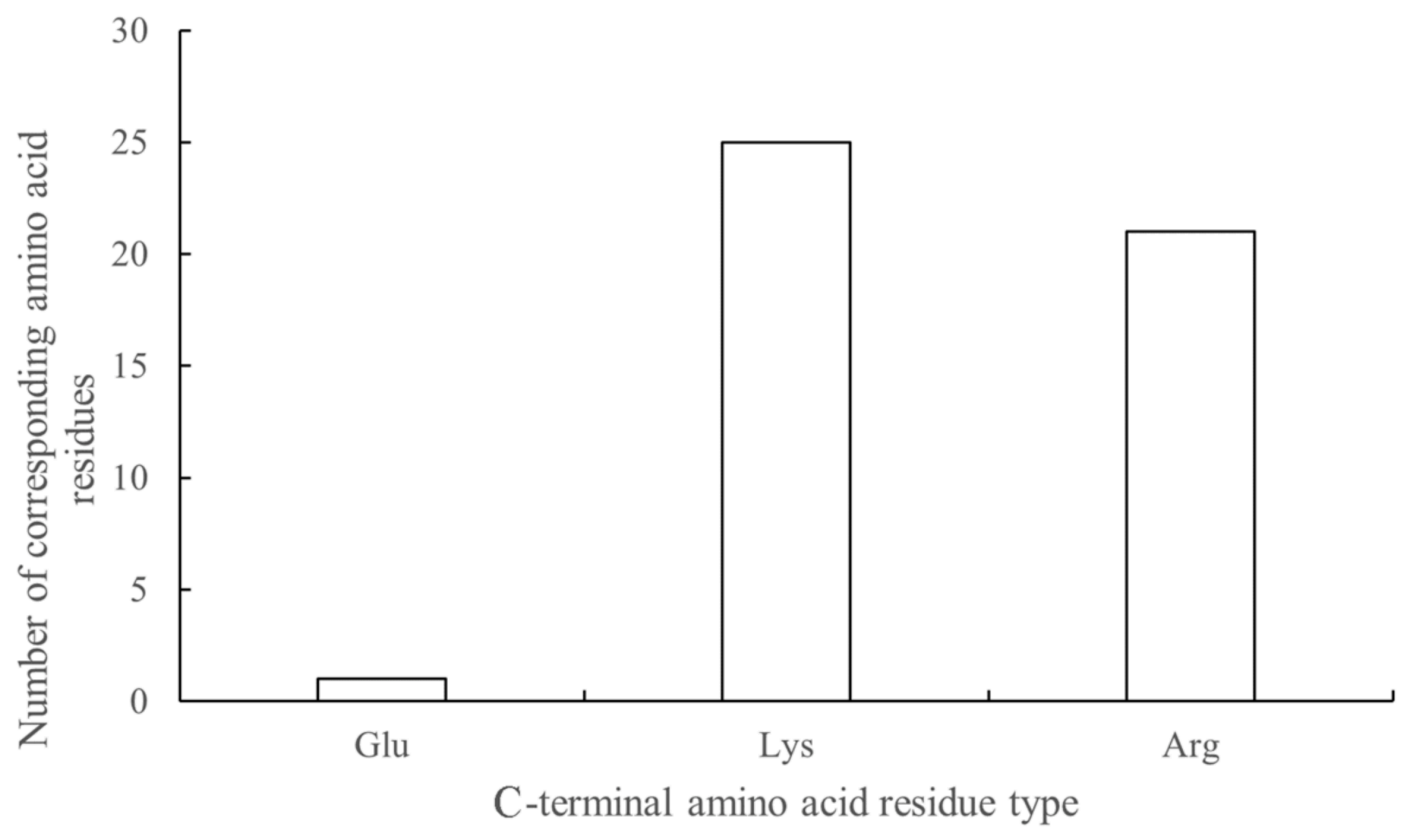
Types of amino acid residues at C-terminal of turtle meat peptides.

**Figure 7:**
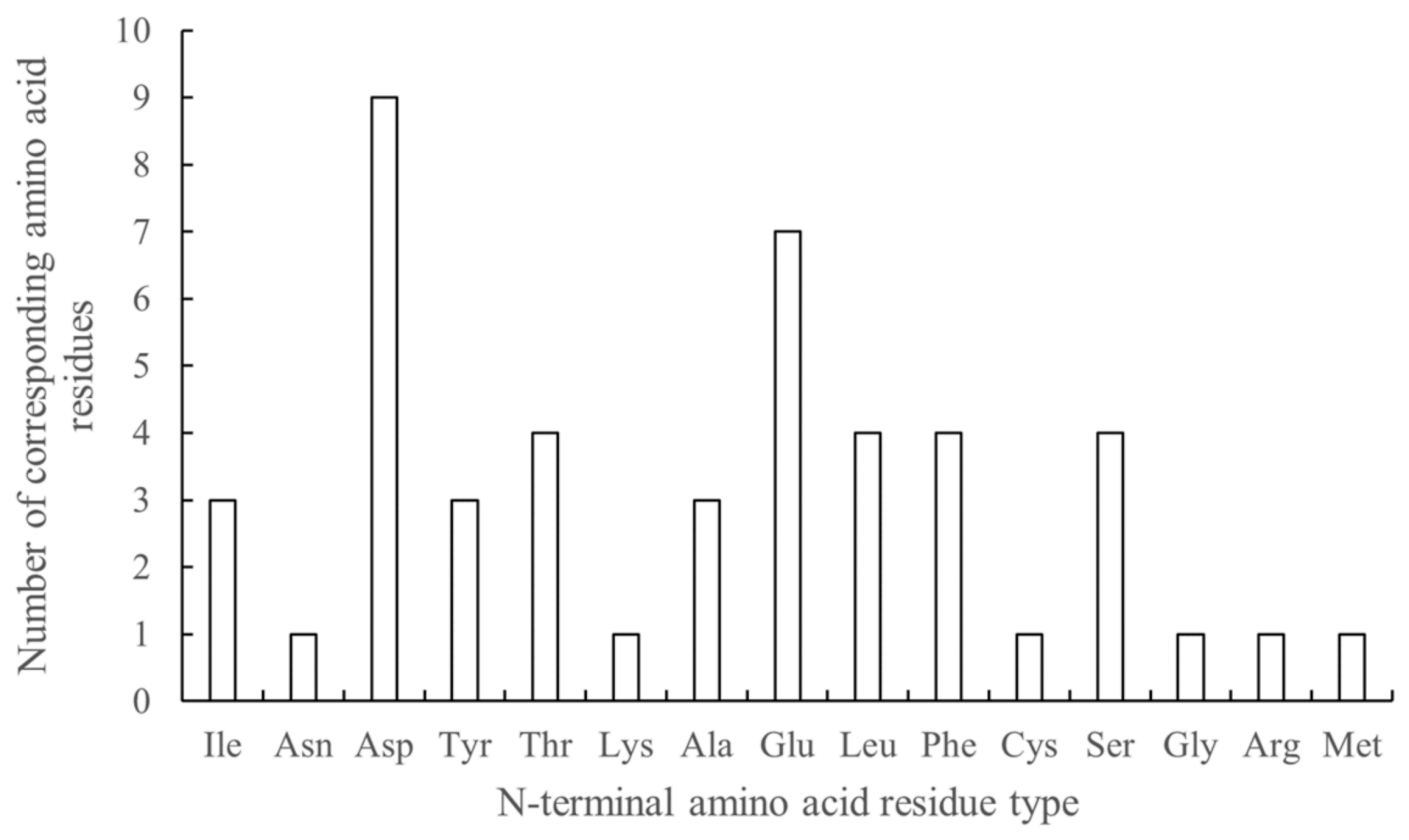
Types of amino acid residues at N-terminal of turtle meat peptides.

The N/C-terminal amino acid residues of the turtle meat-derived peptide segment are shown in Figure 6 and Figure 7. The N/C-terminal amino acid residues of peptides are important structural characteristics that affect their biological activity. The N-terminal amino acid residues of the peptides were mainly negatively charged Asp and Glu, which could easily bind to Zn^2+^ in the active center of ACE, enhancing the ACE inhibitory activity of the peptide [45]. Papain has broad substrate specificity and can hydrolyze peptide bonds at multiple sites. The C-terminal amino acid residues were mainly Lys and Arg, which might be related to the enzymatic hydrolysis characteristics of papain [46]. Previous studies on ACE inhibitory peptides with chain lengths exceeding 10 amino acids showed that differences in inhibitory potency were closely associated with terminal residue composition and peptide–ACE binding patterns, further supporting the importance of N/C-terminal characteristics [47]. The structural features of the peptides identified in this study provide a reasonable explanation for their ACE inhibitory activities.

Hydrophobic amino acid residues can enhance the hydrophobic interaction between peptides and ACE, which is conducive to the binding of peptides to the ACE active center. The content of the hydrophobic amino acid residues of ACE inhibitory peptides is generally higher, and the higher the content, the stronger the ACE inhibitory activity of these peptides [48]. Herein, the analysis of the percentage of hydrophobic amino acid residues in the peptides from turtle meat revealed that each peptide had 22.22 to 58.33% hydrophobic amino acids. Although the content of hydrophobic amino acids in each peptide varies greatly, all peptides contain a certain proportion of hydrophobic amino acids, which further confirms the potential ACE inhibitory activity of these peptides and also provides a basis for the subsequent screening of high-activity ACE inhibitory peptides.

### 3.3. Bioinformatic Characterization of Identified Peptides

The results of the bioinformatic analysis of the peptides are shown in Table S2. Peptide Ranker is a commonly used tool for peptide bioactivity prediction, and its score can provide a preliminary reference for judging the potential activity of the peptides, which is widely used in the screening of ACE inhibitory peptides. Generally, the higher the Peptide Ranker score, the higher the probability that the peptide is active [49]. Good water solubility is beneficial to the dissolution and absorption of peptides, and non-toxicity is the basic premise for the subsequent application of the peptides. In this study, 10 peptides with high Peptide Ranker scores, good water solubility, and predicted non-toxicity were selected for solid-phase synthesis, and the synthetic peptides are shown in Table 1. The synthesized peptides had purities greater than 95%, which meets the requirements of subsequent in vitro ACE inhibitory activity detection and can avoid the interference of impurities in the experimental results, ensuring the reliability of the experiment.

The integration of bioinformatic tools in this study improved the efficiency of the screening of the active peptides. A similar screening strategy has been applied in previous studies, such as the screening of ACE inhibitory peptides from stout beer [50], where Peptide Ranker, water solubility, and ADMET-related properties were jointly considered to prioritize candidates for synthesis and validation. Such comprehensive screening can reduce the number of unnecessary synthesis and in vitro experiments, improve screening efficiency, and avoid resource waste on candidates with poor solubility or potential toxicity. However, the limitations of in silico analysis should be acknowledged. Computational prediction can optimize candidate selection, but it cannot fully replicate the complexity of actual enzymatic hydrolysis. In silico proteolysis does not ensure that the peptides generated computationally can be reproduced experimentally, as proteins may not be completely susceptible to proteolytic cleavage [51]. Thus, experimental validation remains indispensable.

### 3.4. Results of In Vitro ACE Inhibitory Activity of Peptide Fragments

The ACE inhibitory activity of the peptides in vitro is shown in Table 2. Only three of the synthetic peptides showed ACE inhibitory activity. Peptides DK-17, FR-15(C), and YK-17 exhibited ACE inhibitory activity at a concentration of 1.0 mg/mL, with YK-17 showing the strongest ACE inhibitory activity.

The ACE inhibitory activity of YK-17 was further evaluated by determining its IC_50_ value. For this purpose, YK-17 was tested at concentrations of 0.125, 0.25, 0.5, 1, and 2 mg/mL, with all experiments performed in triplicate. A concentration-dependent increase in inhibitory activity was observed for the tested samples. According to the dose–response fitting results, the IC_50_ values was calculated as 479.3 μM for YK-17.

The structural features of the peptides identified in this study provide a reasonable explanation for their ACE inhibitory activities. YK-17, which showed stronger ACE inhibitory activity, contains several residues frequently associated with ACE inhibitory peptides, including aromatic residues Tyr and Trp, the Pro residue, and hydrophobic residues such as Val and Ile. These sequence features may favor hydrophobic interactions with the ACE active pocket and thereby contribute to its inhibitory activity. In contrast, although GK-18 also contains some hydrophobic residues, including Leu, Ala, Ile, and Trp, it is relatively enriched in acidic residues such as Asp and Glu, which are distributed throughout the sequence. This sequence characteristic may influence its binding compatibility with ACE and may partly explain why GK-18 did not exhibit evident ACE inhibitory activity under the tested conditions.

Compared with several reported ACE inhibitory peptides from the BIOPEP-UWM database, including YGNWLYK, YGKPVAVPAR, YQEPVLGPVRGPFPIIV, YMGLDLK, and YETGNGIK [32], YK-17 exhibits a distinct sequence pattern. A common feature shared by YK-17 and all five reference peptides is the presence of Tyr at the N-terminus, which has frequently been associated with enhanced ACE inhibition. Aromatic residues such as Tyr are considered favorable for interaction with the ACE active pocket because they can participate in hydrophobic contacts and stabilize peptide–enzyme binding. In YK-17, this effect may be further reinforced by the presence of Trp at the fourth position, since aromatic and bulky hydrophobic residues are often regarded as important contributors to ACE inhibitory potency. In this respect, the N-terminal region of YK-17 may provide a favorable recognition element for enzyme binding.

Peptide length is another factor worth considering. Most reported ACE inhibitory peptides are relatively short, which is generally advantageous for entering the catalytic channel of ACE and establishing productive interactions with key residues in the active site. In contrast, YK-17 is substantially longer than YGNWLYK, YGKPVAVPAR, YMGLDLK, and YETGNGIK and is more comparable only to the longer peptide YQEPVLGPVRGPFPIIV. This suggests that the ACE inhibitory activity of YK-17 may not be governed solely by peptide length but also by the distribution of activity-related residues within the sequence.

The residue composition of YK-17 also differs noticeably from that of many typical hydrophobic ACE inhibitory peptides. In addition to Tyr and Trp, YK-17 contains several Glu residues and only a limited number of strongly hydrophobic amino acids, giving the peptide a relatively polar and partially acidic character. This feature is distinct from peptides such as YGKPVAVPAR and YQEPVLGPVRGPFPIIV, which are richer in hydrophobic and aliphatic residues and may therefore fit more closely with the conventional view that ACE inhibitory peptides favor hydrophobic side chains, particularly near the C-terminus [52]. Nevertheless, the activity of YK-17 indicates that a highly hydrophobic sequence is not an absolute requirement for ACE inhibition. Instead, the acidic residues in YK-17 may participate in hydrogen bonding or electrostatic interactions with residues around the ACE binding pocket, while the scattered hydrophobic residues, including Trp, Val, and Ile, may provide local anchoring points that support stable binding.

The positional distribution of key residues in YK-17 may also be relevant to its activity. Previous studies [53] have shown that ACE inhibitory peptides often contain aromatic, branched-chain, or Pro residues at the C-terminal region, where they can interact favorably with the S1, S1’, and S2 subsites of ACE. YK-17 does not possess a strongly hydrophobic or Pro-rich C-terminus, ending instead with the relatively polar sequence PQK. From the perspective of classical ACE inhibitory peptide rules, this would not be considered an optimal C-terminal pattern. Therefore, the observed activity of YK-17 suggests that its inhibitory effect may rely less on a canonical hydrophobic C-terminal tail and more on cooperative interactions contributed by the N-terminal Tyr, the internal Trp residue, the Val-rich middle segment, and the conformational influence of Pro and Gly residues within the sequence.

In addition, the presence of Pro at the fifth position and Gly residues in the sequence may influence the local conformation of YK-17. Proline can impose backbone constraints and promote turn-like structures, whereas glycine can enhance local flexibility. Such a combination may help the peptide adopt a binding conformation compatible with the ACE active cleft. These observations suggest that ACE inhibition by YK-17 may depend on cooperative contributions from multiple regions of the peptide, and they provide a rationale for molecular docking studies to identify the sequence elements most critical for activity.

### 3.5. Effects of Synthesized Peptides on HSC-T6 Cell Viability

HSC-T6 cells are a commonly used cell model for hepatic fibrosis research, and changes in HSC-T6 cell viability can serve as a preliminary indicator for evaluating the potential anti-hepatic fibrosis activity of peptides in vitro. The inhibitory effect of the different peptides on the viability of HSC-T6 cells is shown in Table 3. Except for DK-17, all the other peptides exerted different inhibition effects on the viability of HSC-T6 cells. Notably, cell viability gradually declined with the prolongation of the action time of peptides GK-18, FR-15(C), DK-12, IE-9, and LR-16 on the HSC-T6 cells. The enhancement in the inhibitory effect with the prolongation of action time may be due to the continuous accumulation of the peptides in HSC-T6 cells, which leads them to further exert their regulatory effect on cell viability. GK-18 exerted the strongest inhibitory effect on the growth of HSC-T6 cells, with cell viability gradually decreasing when the action time was prolonged up to 48 h. The effect of YK-17 on the growth of HSC-T6 cells was also observed at 24 h, and the effect of YK-17 on HSC-T6 cell viability was second to that of GK-18. These results provide preliminary evidence that GK-18 may exert potential antifibrotic effects in vitro through reducing HSC-T6 cell viability, and further studies are required to confirm their anti-hepatic fibrosis activity and underlying mechanisms.

This finding is in line with previous studies reporting that natural peptides can effectively inhibit the viability of activated HSC-T6 cells. For example, collagen-derived peptides from *Eupolyphaga sinensis* have been shown to suppress HSC-T6 cell viability in a dose-dependent manner [54], suggesting that animal-derived peptides are a viable source of candidates for anti-hepatic fibrosis applications. The time-dependent inhibitory effect of the peptides in this study is also consistent with existing studies. For instance, a study [55] on gallic acid demonstrated that it could inhibit HSC-T6 cell viability in a time-dependent manner. The underlying mechanism of this time-dependent effect was speculated to be the continuous accumulation of gallic acid in HSC-T6 cells, which is consistent with the hypothesis proposed in this study.

To better understand the structural features associated with the anti-HSC-T6 cell activity of GK-18, three previously reported antifibrotic peptides, GLSKGCFGLKLDRIGSMSGLGC [56], GPPGVPGPGPL [57], and GAGPHGG [58], were taken as references. GAGPHGG, a peptide isolated from turtle shell (Carapax trionycis), has been reported to inhibit the viability of HSC-T6 cells and exert anti-hepatic fibrosis activity. It provides a structural reference for understanding the activity of GK-18. Although their sequences differ substantially, these peptides all display regulatory effects on fibrosis-related cellular responses. GAGPHGG has been shown to inhibit HSC-T6 cell proliferation in a time- and dose-dependent manner and to markedly downregulate alpha-SMA and TGF-beta1 expression. GPPGVPGPGPL exhibited significant anti-hepatic fibrosis activity in TGF-beta1-induced LX-2 cells, possibly by competitively disrupting the interaction between TGF-beta1 and its receptor through hydrogen bond formation. Meanwhile, GLSKGCFGLKLDRIGSMSGLGC was reported to alleviate pulmonary fibrosis and improve survival in mice by suppressing TGF-beta signaling and myofibroblast differentiation. These findings provide a useful context for interpreting the structural characteristics and possible mode of action of GK-18.

Compared with these three reported peptides, GK-18 displays several notable structural features. First, although all these peptides begin with Gly, the contribution of the N-terminal Gly to antifibrotic activity is likely to depend on the surrounding residue environment rather than on the terminal residue alone. The presence of Gly at the N-terminus may reduce steric hindrance and enhance conformational flexibility, thereby facilitating target recognition and contributing to the inhibitory effect on HSC-T6 cell viability. This explanation remains speculative and requires further validation. Second, GK-18 is longer than GAGPHGG and GPPGVPGPGPL but shorter than GLSKGCFGLKLDRIGSMSGLGC, suggesting that its activity may not be determined solely by peptide length. Shorter peptides may penetrate the cell membrane more readily and interact more efficiently with intracellular or membrane-associated targets, thereby reducing cell viability more effectively. Although shorter peptides such as GAGPHGG may more readily access intracellular or receptor-associated targets, the bioactivity of GK-18 implies that an appropriate chain length combined with a favorable residue arrangement may also support antifibrotic activity.

More importantly, the amino acid composition of GK-18 differs substantially from that of the three reference peptides. GAGPHGG and GPPGVPGPGPL are rich in Gly and Pro, which are typically associated with high backbone flexibility and may favor close fitting to protein-binding interfaces. By contrast, GK-18 is enriched in acidic residues, containing multiple Asp and Glu residues, and has only one basic residue at the C-terminus. This sequence characteristic suggests that GK-18 is unlikely to behave as a typical cationic membrane-active peptide; instead, its anti-HSC-T6 activity may be associated with specific electrostatic and hydrogen bond interactions with fibrosis-related target proteins or receptors. In this respect, GK-18 may be closer to GPPGVPGPGPL in its putative mode of action, as its sequence characteristics do not support a typical nonspecific membrane-disruptive mechanism but instead suggest a greater likelihood of specific interference with fibrosis-related molecular recognition events, possibly involving the TGF-beta signaling axis.

Furthermore, GK-18 contains several hydrophobic residues, including Leu, Ile, Val, and Trp, which may provide a complementary hydrophobic interaction surface for binding to target proteins. In particular, the C-terminal Trp and Lys residues may contribute to the activity of GK-18, as Trp is frequently associated with hydrophobic or interfacial binding interactions, whereas Lys may help stabilize target recognition through local electrostatic effects. In contrast, the cysteine-containing peptide GLSKGCFGLKLDRIGSMSGLGC is amphiphilic and relatively rich in basic residues, indicating a more complex interaction pattern with signaling-related targets. Therefore, although GK-18 shares inhibitory activity against HSC-T6 cell viability with these reported peptides, its sequence characteristics suggest that it represents a structurally distinct bioactive peptide.

Taken together, the comparative analysis suggests that the anti-HSC-T6 activity of GK-18 may arise from a combination of N-terminal Gly, an appropriate peptide length, a hydrophobic residue framework, and a unique acidic amino acid distribution. Unlike the Gly- and Pro-dominant peptides GAGPHGG and GPPGVPGPGPL or the basic cysteine-containing peptide GLSKGCFGLKLDRIGSMSGLGC, GK-18 possesses a characteristic acidic sequence context, which may favor the selective modulation of fibrosis-associated signaling pathways, especially those related to TGF-β. These observations provide a plausible structural explanation for the inhibitory effect of GK-18 on HSC-T6 cell viability and support further mechanistic and residue substitution studies to clarify the sequence features underlying its activity.

### 3.6. Molecular Docking Analysis

The ACE C-terminal domain binding site consists of three active pockets (S1, S2, and S1’) and a zinc-binding site. The S1 pocket contains three reported key residues, including Ala354, Glu384, and Tyr523. The S2 pocket has five reported key residues, Gln281, His353, Lys511, His513, and Tyr520, and the S1’ pocket has one reported key residue, Glu162. The zinc-binding domain contains three synergistic residues: His383, His387, and Glu411 [53,59]. Additionally, Arg381, Tyr369, Lys489, Tyr498, Gln259, Ser39, Asp44, Ser199, and Arg500 have also been reported as key residues in the active site of the ACE N-terminal structural domain [60]. The identification of key residues in the N-/C-terminal structural domains of ACE provides an important basis for analyzing the binding mechanism between peptides and ACE, and the specific interacting residues may vary depending on the peptide ligand.

It should be noted that the predicted binding energy and interaction pattern obtained from molecular docking cannot be directly equated with the experimentally determined IC_50_ values. Molecular docking provides a structure-based and static prediction of the possible binding mode between peptides and ACE, whereas IC_50_ reflects the overall inhibitory potency measured under specific experimental conditions. Nevertheless, docking analysis can still provide useful mechanistic clues by revealing the potential binding sites of peptides and their interactions with key amino acid residues of ACE, including hydrogen bonding, hydrophobic interactions, electrostatic interactions, and possible coordination with Zn^2+^. These structural features may help to explain the differences in ACE inhibitory activity among peptides.

Binding energy is an important indicator for evaluating the stability of peptide–receptor binding. Generally, the lower the binding energy, the more stable the binding between the peptide and the receptor. Herein, the binding energies of YK-17 and N/C-ACE were lower than ‒5.0 kJ·mol^−1^ (Table 4), suggesting that the binding between the peptide with both C-ACE and N-ACE is relatively stable [61]. In addition, various interaction forces exist between ligands and receptors, including hydrogen bonding force, hydrophobic interaction, van der Waals force, π-bonding and electrostatic force, among which hydrogen bonding is the most important non-covalent interaction in the binding process of biologically active peptides with ACE [62].

**Figure 8:**
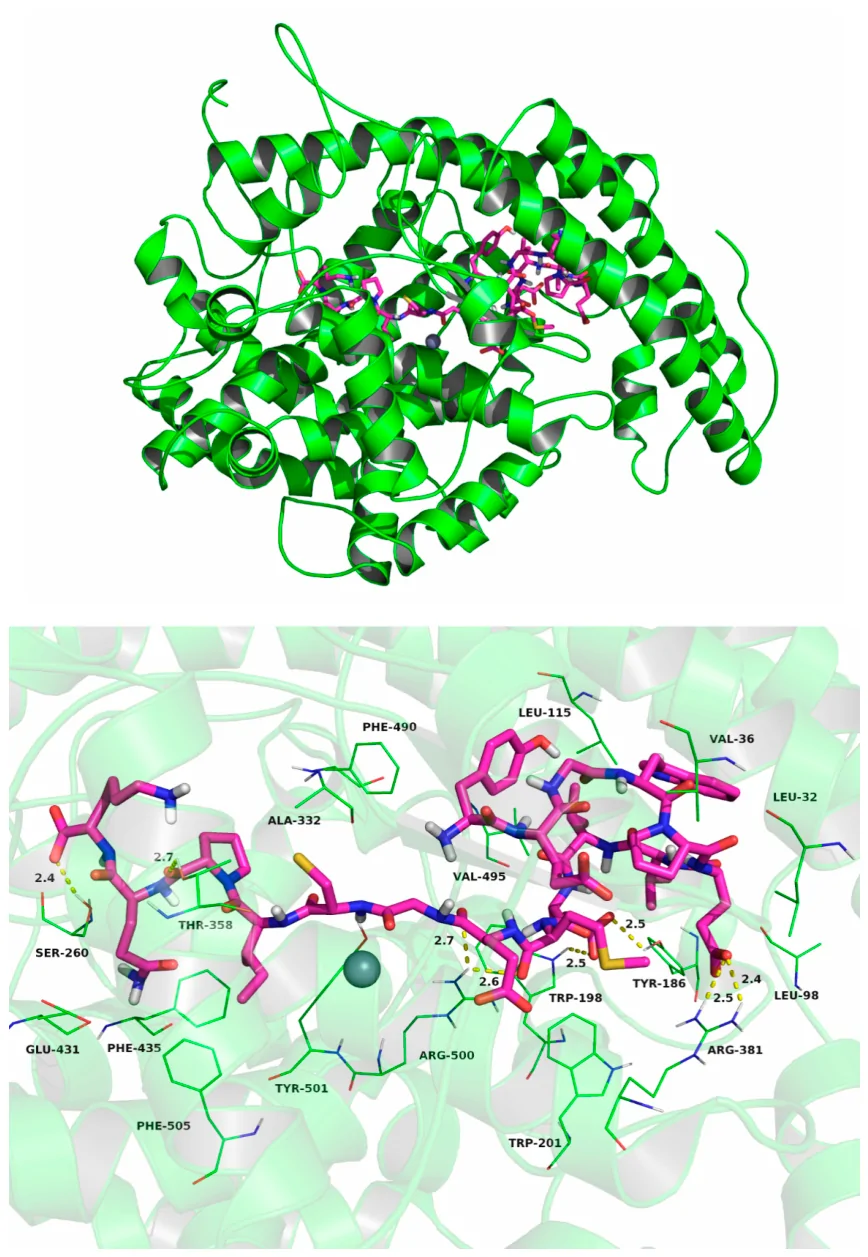
Overall and detailed view of YK-17 docking with N-ACE.

Peptide YK-17 bound to the N-ACE binding pocket in a compact conformation (Figure 8). Peptide YK-17 was in a hydrophobic luminal pocket consisting of amino acid residues, Leu-32, Val-36, Leu-98, Leu-115, Trp-198, Trp-201, Ala-332, Phe-435, Phe-490, Val-495 and Phe-505, forming a strong hydrophobic interaction. Further analysis revealed that peptide YK-17 formed anion–π interactions with amino acid residues Tyr-186 and Trp-198, and CH-π interactions also existed with amino acid residue Phe-490. Importantly, peptide YK-17 formed multiple hydrogen bonding interactions with amino acid residues Ser-260 (2.4 Å), Thr-358 (2.4 Å), Arg-500 (2.6 Å and 2.7 Å), Trp-198 (2.5 Å), Tyr-186 (2.5 Å), and Arg-381 (2.4 Å and 2.5 Å).

**Figure 9:**
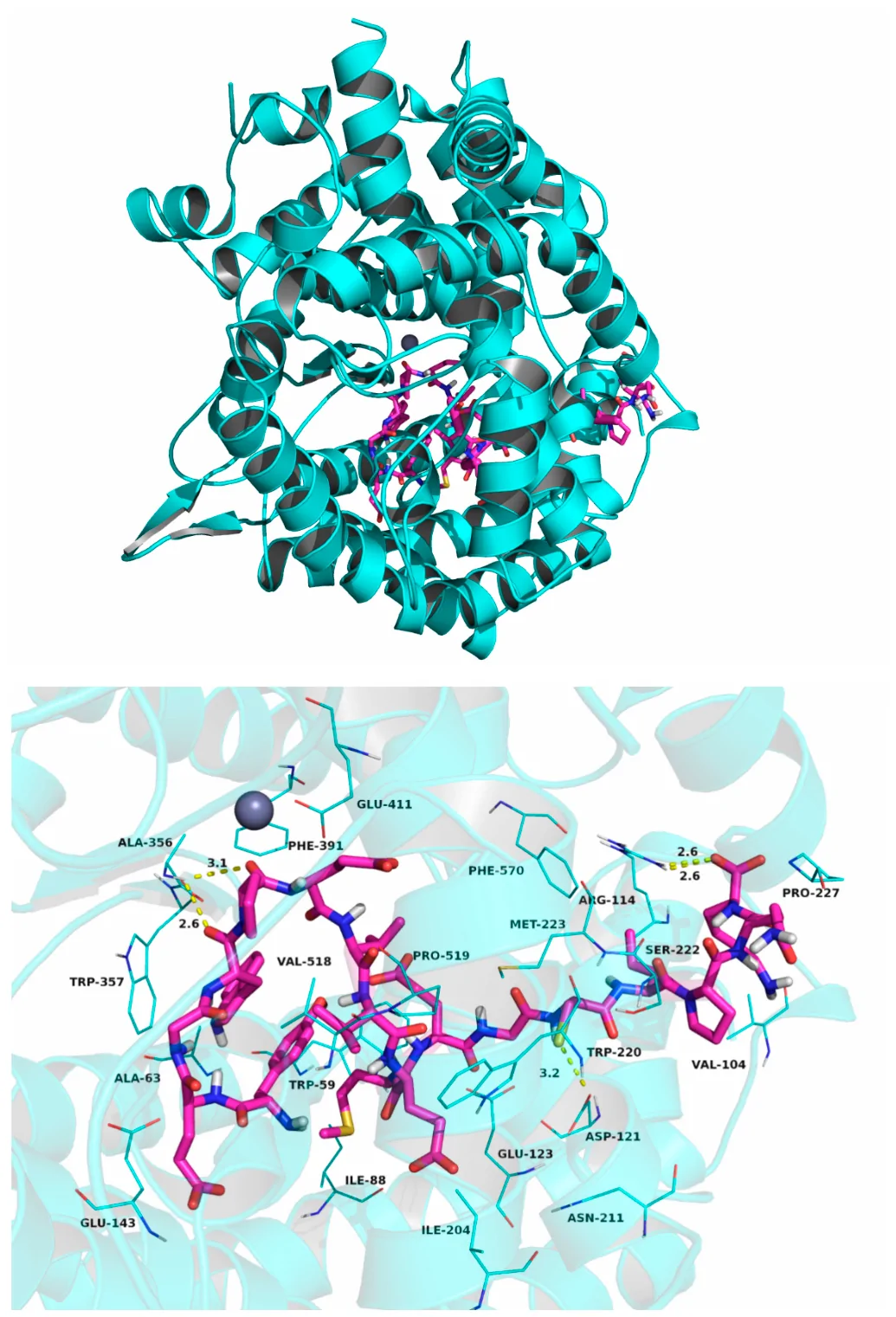
Overall and detailed view of YK-17 docking with C-ACE.

The docking results of peptide YK-17 with C-ACE are shown in Figure 9. Peptide YK-17 was in the hydrophobic region consisting of amino acid residues Trp-59, Ala-63, Ile-88, Val-104, Ile-204, Trp-220, Met-223, Pro-227, Ala-356, Trp-357, Phe-391, Val-518, Pro-519 and Phe-570, which formed a strong hydrophobic interaction. Notably, peptide YK-17 formed an anion–π interaction with amino acid residue Trp-59, an electrostatic interaction with amino acid residue Arg-114, and a π-π stacking interaction with amino acid residue Trp-347. More importantly, peptide YK-17 formed triple hydrogen bonding interactions with amino acid residues Asp-121 (3.2 Å), Arg-114 (2.6 Å and 2.6 Å) and Ala-356 (2.6 Å and 3.1 Å).

In summary, hydrophobic interaction and hydrogen bonding forces existed between YK-17 with different structural domains of the ACE N/C terminal. In addition, the hydrogen bonds between peptides and N-ACE were stronger than the hydrogen bonds formed with C-ACE, suggesting that YK-17 have stronger hydrogen bonding forces with N-ACE. Hydrogen bonding is an important factor in stabilizing peptide–enzyme complexes. Therefore, these docking results suggest that domain-related differences in peptide–ACE interactions may be involved in the activities of the peptides.

In the present study, YK-17 exhibited stronger ACE inhibitory activity, while its docking results suggested a more favorable interaction with the C-domain of ACE. This may be partly related to the more important role of the ACE C-domain in catalytic activity. These results suggest that the biological activities of the peptides may depend not only on predicted binding energy but also on multiple factors such as peptide sequence, hydrophobic interactions and hydrogen bonding patterns. Therefore, further studies are needed to better explain the relationship between molecular docking analysis and experimentally measured IC_50_ values.

### 3.7. Predicted Gastrointestinal Digestion Characteristics and Potential Bioactivity of YK-17 and GK-18

The digestive stability of the two target peptides, YK-17, which exhibited strong ACE inhibitory activity, and GK-18, which showed a marked inhibitory effect on HSC-T6 cell viability, was further preliminarily assessed by in silico enzymatic digestion. YK-17 was predicted to remain intact after pepsin and trypsin treatment but to be cleaved by chymotrypsin into three smaller peptides. Analysis using the BIOPEP-UWM database showed that none of these fragments had been previously recorded; however, all three peptides were predicted to be potential ACE inhibitory peptides, suggesting that the digestion products of YK-17 may retain potential ACE inhibitory activity after simulated gastrointestinal digestion. In contrast, GK-18 was predicted to be resistant to trypsin, whereas pepsin and chymotrypsin cleaved it into five peptides. Among these, only one fragment matched a known ACE inhibitory peptide in the BIOPEP-UWM database, while the other four fragments were neither recorded in the database nor predicted to exhibit ACE inhibitory activity. In addition, cell-penetrating peptide prediction suggested that these four peptides might have limited cellular uptake potential, indicating that the inhibitory potential of GK-18 toward HSC-T6 cell proliferation may be reduced after simulated gastrointestinal digestion. Nevertheless, this prediction requires further experimental validation.

## 4. Conclusions

A total of 47 ACE inhibitory peptides, composed of 9–23 amino acid residues with molecular weights from 1000 to 2600 Da, were identified from turtle meat, three of which exhibited ACE inhibitory activity. The N-terminal amino acid residues showed considerable diversity, among which Asp and Glu were relatively frequent, while the C-terminal residues were predominantly Lys and Arg. Moreover, hydrophobic amino acid residues accounted for a relatively high proportion of the peptide sequences. Among these peptides, 34 peptides were non-toxic and showed good water solubility. The peptide YK-17 showed potent ACE inhibitory activity with an IC_50_ value of 479.3 μM; peptide GK-18 showed the strongest inhibitory activity against HSC-T6 cell viability. An analysis of molecular interactions showed that YK-17 bound to the N-terminal and C-terminal domains of ACE and formed more hydrogen bonds with N-ACE than with C-ACE. The shorter peptides showed stronger inhibitory effects on the viability of HSC-T6 cells. Possessing Gly at the N-terminus may be an important characteristic for stronger inhibitory effects on HSC-T6 cell viability.

## Figures and Tables

**Table 1:** Information about the synthesized peptides.

Peptide Sequence	Name	Length	Molecular Mass ^1^
DDDIAALVVDNGSGMCK	DK-17	17	1722.89
FDEILEASDGIMVAR	FR-15(C)	15	1665.86
IPAPDDPHE	IE-9	9	990.02
DLDVAILVGSMPR	DR-13	13	1385.62
YEGWPEVVEMEGCIPQK	YK-17	17	1994.24
GLCVDALIELSDENADWK	GK-18	18	1991.17
FEVIEFDDGSGSVLR	FR-15(N)	15	1669.82
EDLLAEAWKEDNSLESPR	ER-18	18	2102.21
LEYISEAAFEGLVNLR	LR-16	16	1824.03
DICNDVLSLLEK	DK-12	12	1361.56

^1^ Molecular weight refers to the measured value.

**Table 2:** ACE inhibitory activity of peptides (1.0 mg/mL) in vitro (±S, n = 3, *p* < 0.0001).

Name	ACE Inhibition Rate %
DK-17	4.57 ± 0.64
FR-15(C)	9.63 ± 1.49
IE-9	-
DR-13	-
YK-17	57.69 ± 2.50
GK-18	-
FR-15(N)	-
ER-18	-
LR-16	-
DK-12	-

**Table 3:** Effects of synthesized peptides on viability of HSC-T6 cells (±S, n = 3, *p* < 0.0001).

Peptides	Cell Viability (%)	
	24 h	48 h
DR-13	83.72 ± 2.43	91.22 ± 0.71
GK-18	74.06 ± 3.59	64.60 ± 9.22
FR-15(N)	82.35 ± 1.42	87.20 ± 3.45
FR-15(C)	89.18 ± 0.32	88.87 ± 3.33
DK-12	86.59 ± 1.09	85.51 ± 4.22
LR-16	93.21 ± 0.63	92.44 ± 0.84
ER-18	84.55 ± 3.41	88.40 ± 4.30
YK-17	79.66 ± 0.51	88.10 ± 4.07
IE-9	81.25 ± 0.27	80.46 ± 2.78
DK-17	105.55 ± 5.45	99.47 ± 0.64

**Table 4:** Molecular docking results of peptide YK-17 with C- and N-domains of ACE.

Peptide	Binding Energy (kJ·mol^−1^)	
	C-ACE	N-ACE
YK-17	−10.4	−9.2

## Data Availability

The original contributions presented in this study are included in the article/Supplementary Material. Further inquiries can be directed to the corresponding author.

## Supplementary Materials

The following supporting information can be downloaded at https://www.mdpi.com/article/10.3390/foods15162805/s1, Figure S1. Ten screened peptides from turtle meat by HPLC-MS/MS. (Note: MS/MS spectra of (1) DDDIAALVVDNGSGMCK, (2) FDEILEASDGIMVAR, (3) IPAPDDPHE, (4) DLDVAILVGSMPR, (5) YEGWPEVVEMEGCIPQK, (6) GLCVDALIELSDENADWK, (7) FEVIEFDDGSGSVLR, (8) EDLLAEAWKEDNSLESPR, (9) LEYISEAAFEGLVNLR, (10) DICNDVLSLLEK; Table S1. LC-MS/MS identification of peptides from the active fractions of turtle meat.; Table S2. Predicted results of the bioinformatics analysis for the 47 peptides.
