# Supplementary material for: The Isolation and Characterization of ACE Inhibitory Peptides from Pelodiscus sinensis Wiegmann Meat and Their Effects on the Viability of HSC-T6 Cells
Source: Foods. 2026 Aug 11;15(16):2805. doi: 10.3390/foods15162805 (PMC13512709; doi:10.3390/foods15162805)
Supplement: Supplementary file 1 [file foods-15-02805-s001.zip › foods-4399393-supplementary.pdf]

Supporting Information

Isolation and Characterization of ACE Inhibitory Peptides from *Pelodiscus sinensis* Wiegmann Meat and Their Effects on the Proliferation of HSC-T6 Cells

Fangze Jiao, Xueqin Sun, Tianfeng Zhang, Xun Li, Guanglan Zheng, Lini Que, Yaming Liang and Pengying Liao *

College of Pharmacy, Guangxi University of Chinese Medicine, Nanning 530200, China; jiaofangze2024@stu.gxtcmu.edu.cn (F.J.); sun_yue1998@163.com (X.S.); zhangtianfeng2023@stu.gxtcmu.edu.cn (T.Z.); lixun2024@stu.gxtcmu.edu.cn (X.L.); zgl2875161173@outlook.com (G.Z.); qln18176924139@outlook.com (L.Q.); 18177508352@163.com (Y.L.)

* Correspondence: gxlpy@163.com

Present address: No. 13, Wuhe Avenue, Nanning Guangxi 530200, China

Contents

Figure S1. Ten screened peptides from turtle meat by HPLC-MS/MS. (Note: MS/MS spectra of (1) DDDIAALVVDNGSGMCK, (2) FDEILEASDGIMVAR, (3) IPAPDDPHE, (4) DLDVAILVGSMPR, (5) YEGWPEVVEMEGCIPQK, (6) GLCVDALIELSDENADWK, (7) FEVIEFDDGSGSVLR, (8) EDLLAEAWKEDNSLESPR, (9) LEYISEAAFEGLVNLR, (10) DICNDVLSLLEK

Table S1. LC-MS/MS identification of peptides from the active fractions of turtle meat.

Table S2. Predicted results of the bioinformatics analysis for the 47 peptides.

(1)

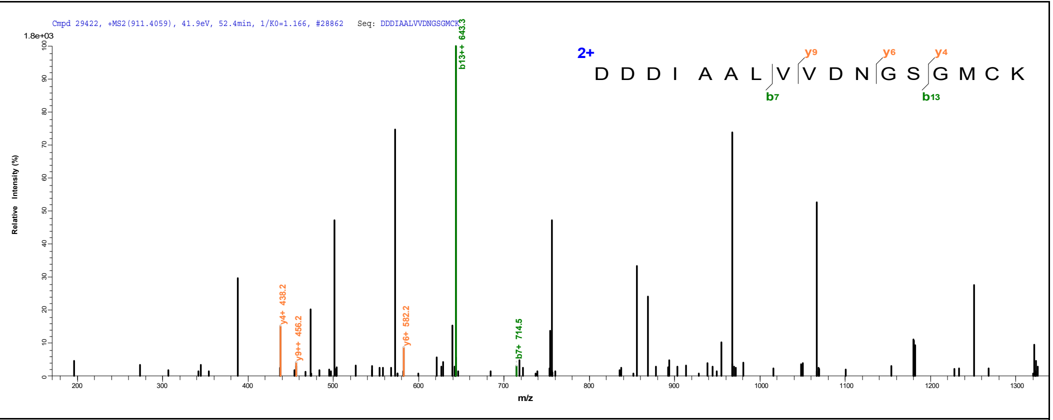
Cmpd 29422, +MS2(911.4059), 41.9eV, 52.4min, 1/K0=1.166, #28862 Seq: DDDIAALVVDNGSGMCK
1.8e+03
Relative Intensity (%)
m/z
2+
D D D I A A L V V D N G S G M C K
y9 y6 y4
b7 b13
y4+ 438.2
y9++ 456.2
y6+ 582.2
b13++ 643.3
b7+ 714.5

(2)

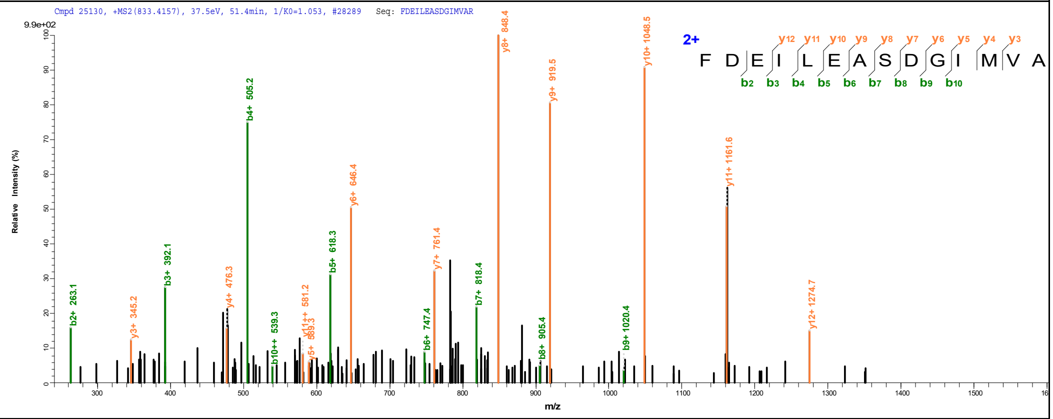
Cmpd 25130, +MS2(833.4157), 37.5eV, 51.4min, 1/K0=1.053, #28289 Seq: FDEILEASDGIMVAR
9.9e+02
Relative Intensity (%)
m/z
2+
F D E I L E A S D G I M V A
y12 y11 y10 y9 y8 y7 y6 y5 y4 y3
b2 b3 b4 b5 b6 b7 b8 b9 b10
b2+ 263.1
y3+ 345.2
b3+ 392.1
y4+ 476.3
b4+ 505.2
b10++ 539.3
y11++ 581.2
b5+ 618.3
y6+ 646.4
b6+ 747.4
y7+ 761.4
b7+ 818.4
y8+ 848.4
b8+ 905.4
y9+ 919.5
b9+ 1020.4
y10+ 1048.5
y11+ 1161.6
y12+ 1274.7

(3)

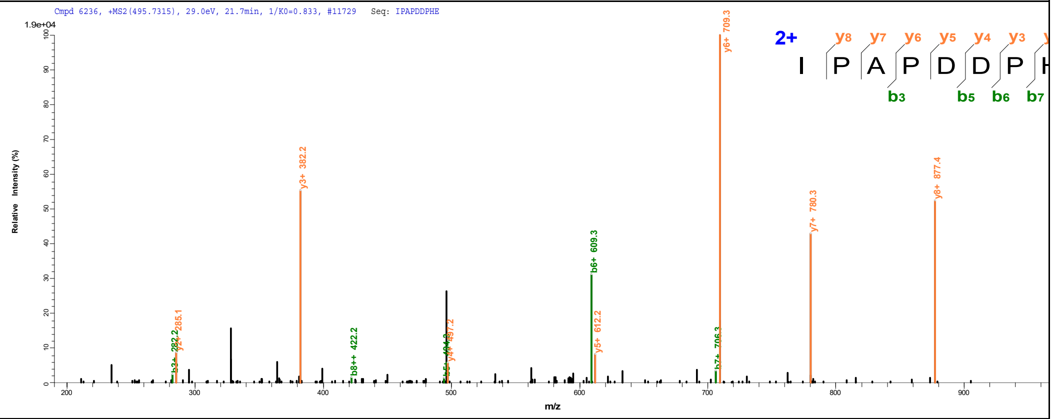
Cmpd 6236, +MS2(495.7315), 29.0eV, 21.7min, 1/K0=0.833, #11729 Seq: IPAPDDPHE
1.9e+04
Relative Intensity (%)
m/z
2+
I P A P D D P
y8 y7 y6 y5 y4 y3
b3 b5 b6 b7
y3+ 382.2
b8++ 422.2
b6+ 609.3
y6+ 709.3
y7+ 780.3
y8+ 877.4

(4)

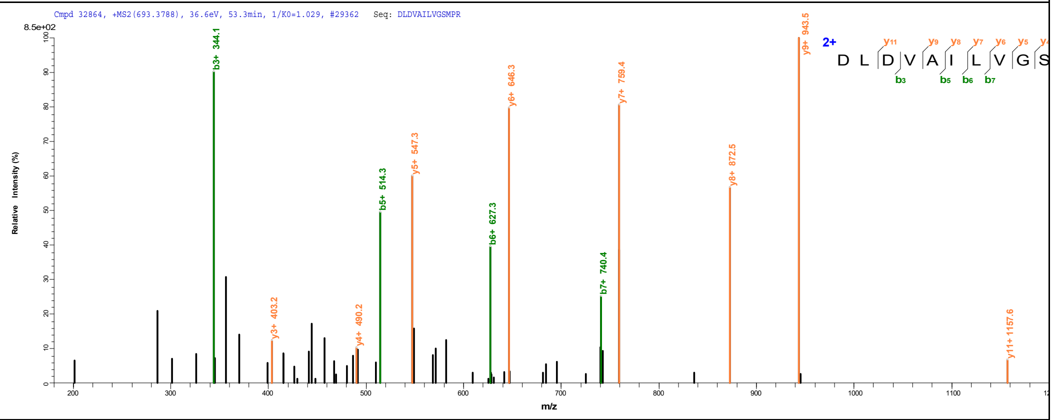
Cmpd 32864, +MS2(693.3788), 36.6eV, 53.3min, 1/K0=1.029, #29362 Seq: DLDVAILVGSMPR
8.5e+02
Relative Intensity (%)
m/z
2+
D L D V A I L V G S
y11 y9 y8 y7 y6 y5
b3 b5 b6 b7
b3+ 344.1
y3+ 403.2
y4+ 490.2
b5+ 514.3
y5+ 547.3
b6+ 627.3
y6+ 646.3
b7+ 740.4
y7+ 759.4
y8+ 872.5
y9+ 943.5
y11+ 1157.6

(5)

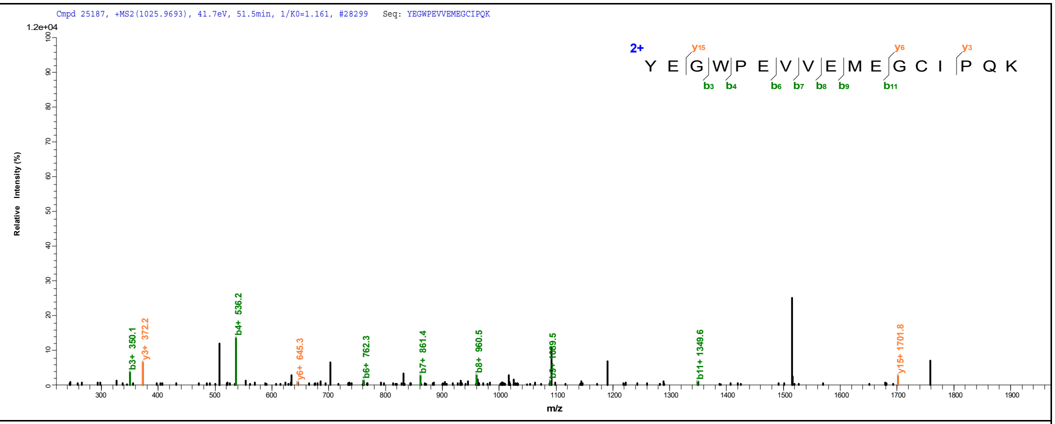
Cmpd 25187, +MS2(1025.9693), 41.7eV, 51.5min, 1/K0=1.161, #28299 Seq: YEGWPEVVEMEGCIPQK
2+
Y E G W P E V V E M E G C I P Q K
Relative Intensity (%)
m/z

(6)

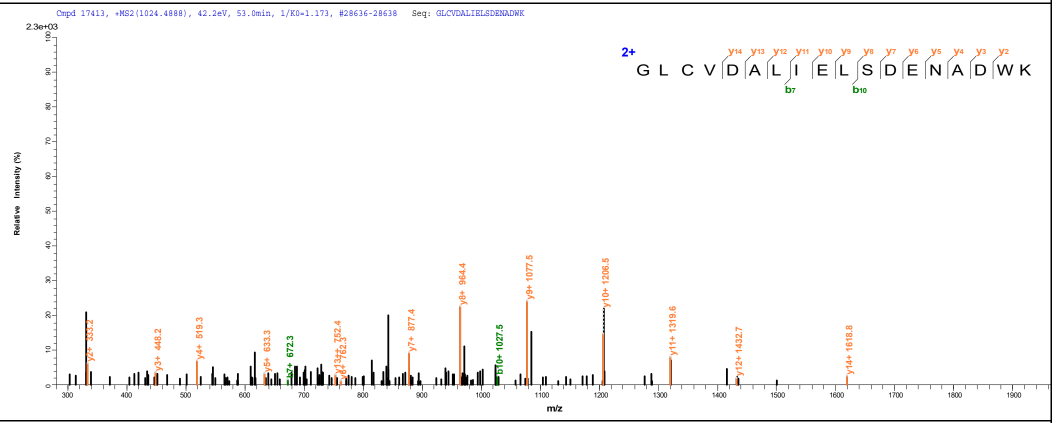
Cmpd 17413, +MS2(1024.4888), 42.2eV, 53.0min, 1/K0=1.173, #28636-28638 Seq: GLCVDALIELSDENADWK
2+
G L C V D A L I E L S D E N A D W K
Relative Intensity (%)
m/z

(7)

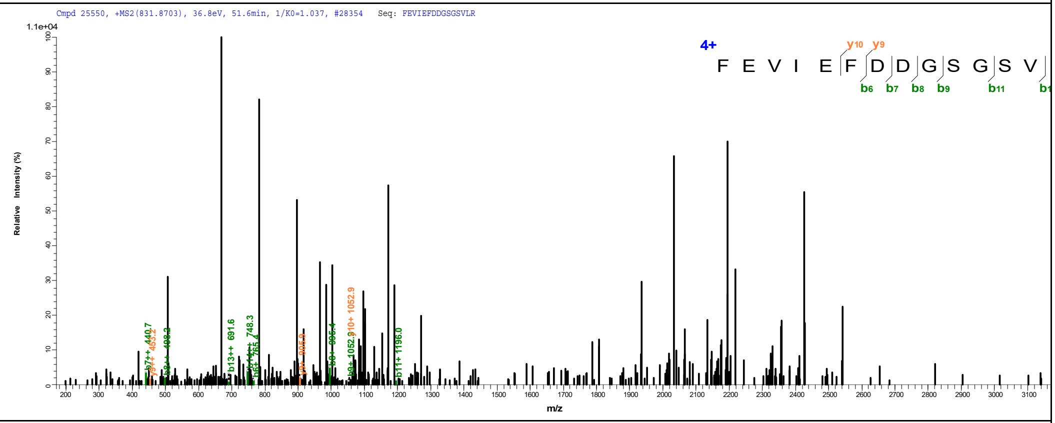
Cmpd 25550, +MS2(831.8703), 36.8eV, 51.6min, 1/K0=1.037, #28354 Seq: FEVIEFDDGSGSVLR
4+
F E V I E F D D G S G S V
Relative Intensity (%)
m/z

(8)

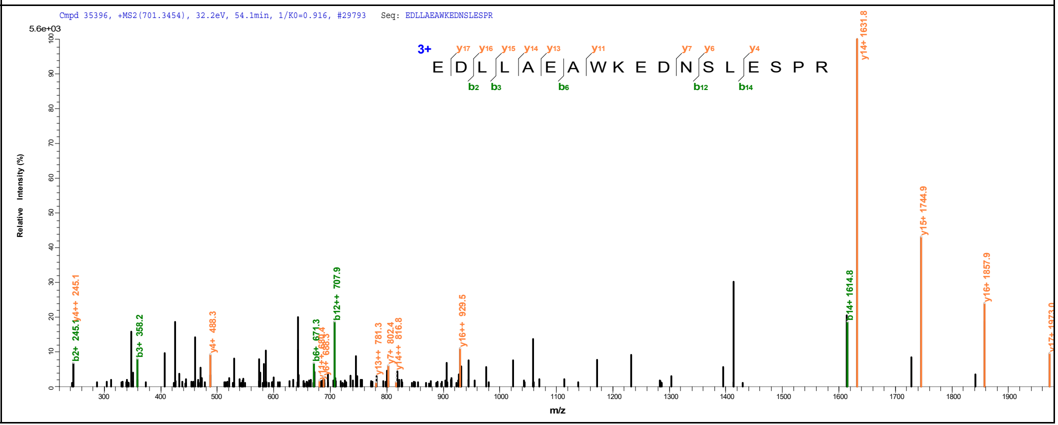
Cmpd 35396, +MS2(701.3454), 32.2eV, 54.1min, 1/K0=0.916, #29793 Seq: EDLLAEAWKEDNSLESPR
3+
E D L L A E A W K E D N S L E S P R
Relative Intensity (%)
m/z

(9)

(10)

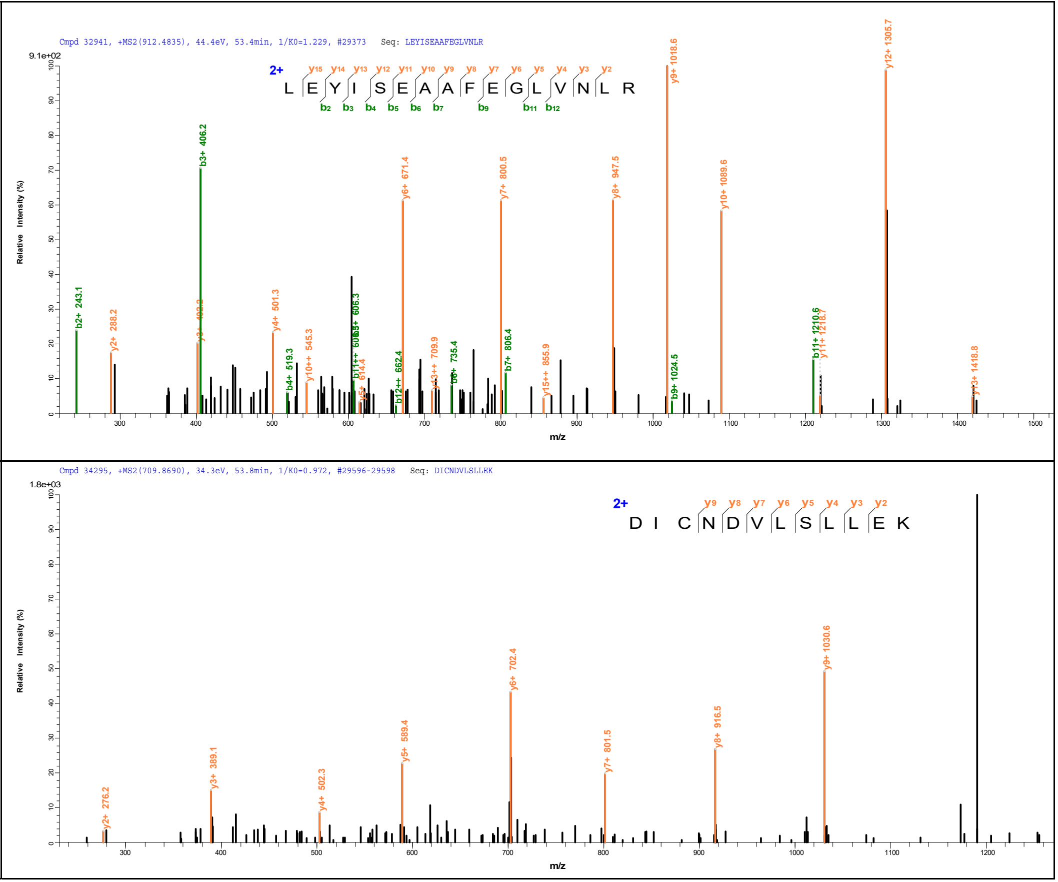

Figure S1

Table S1. LC-MS/MS identification of peptides from the active fractions of turtle meat.

Peptide sequence	Accession number of the source protein	*m/z*	Identif-ication Score
ELENLAAMDLELQK	K7GFN8	1615.8127	302.85
EFEGEEEYLEILGITR	K7F3M3	1925.9258	289.48
FSGSGSGTDFTLTISR	K7FYE6;K7FX95;K7G0P2	1631.7791	211.52
IDTIAADESFTQVDIGDR	K0ISK7;K7FBC9	1964.9327	206.7
DIASGLIGPLLICK	K7F2H6	1468.8323	153.33
TITLEVEPSDTIENVK	K7FWJ8;K7F5N0	1786.9200	151.66
ADQLTEEQIAEFK	K7G387;K7FDQ7	1520.7359	147.02
CLVGEFVSDVLLVPEK	K7GB27;K7GB12	1802.9488	144.51
YEGWPEVVEMEGCIPQK	K7FI48	2049.9176	135.13
EYVLPSFEVILEPSEK	K7GDB0	1877.9662	119.43
DLADELALVDVIEDK	K7F3R5;Q98SL0;K7F3R3	1656.8458	112.95
SYELPDGQVITIGNER	K7F239;K7F0D9;B3GS65;K7FID2;K7FJK5;K7FGA7;K7FF29;K7FDA4;K7F4I9;K7FVI4	1789.8846	111.38
IPAPDDPHE	K7F724	989.44543	108.92
FDEILEASDGIMVAR	K7FL59;K7FL68	1664.8080	103.76
LEYISEAAFEGLVNLR	K7GAI2	1822.9465	102.03
YEELQLTAGR	K7F739K7F6A7	1178.5932	101.82

Peptide sequence	Accession number of the source protein	*m/z*	Identif-ication Score
LYTLVLTDPDAPSR	K7F9Y4	1559.8195	101.45
SQEPVTLDFLDAELENDIK	K7GIH0;K7GIC9	2175.0583	100.98
EEEEVLDQGDFYSLISK	K7FAH0	1999.9262	99.668
TYYIAAVEIEWDYSPNR	K7GBY;K7GBV3	2088.9793	99.589
DLDVAILVGSMPR	K7G8Q8	1384.7384	99.174
DDDIAALVVDNGSGMCK	K7FVI4	1778.7815	98.167
IMELQDILAK	K7FSK3;K7FSH9	1172.6475	98.009
DLYANTVLSGGTTMYPGIADR	K7F239;K7F0D9	2214.0627	94.119
AGTFFWSVVIPHER	K7GCG2;K7GCH5	1644.8413	92.707
TAFDEAIAELDTLSEESYK	K7F7A3	2130.9845	87.727
YTSALTYDGVLVMAETFR	K7G366;K7G377	2035.9925	86.729
FEVIEFDDGSGSVLR	K7G2M8	1668.7995	85.813
TIAVLLDDILQR	K7GA84	1368.7977	85.622
FYPEDVSEELIQDITQR	K7G7K9	2080.9953	85.081
ATVHIQVNDVNEYAPVFK	K7F8Y0;K7F8X6	2043.0425	82.437
LIAELGSTSISNLGFR	K7FI48	1676.9097	81.799
REEEEVLDQGDFYSLISK	K7FAH0	2156.0273	81.578

Peptide sequence	Accession number of the source protein	*m*/*z*	Identif-ication Score
GLCVDALIELSD ENADWK	K7FUA5	2046. 9568	77.696
DICNDVLSLLEK	K7F7A3	1417. 7123	76.255
DSTLIMQLLR	K7F7A3;K7EYU9;K7FXZ6; K7G3H0;K7G1P2;K7EWQ2	1188. 6536	74.486
EITVATGGFIYTG EVVHR	K7GEH5;K7GEI2	1948. 0054	73.502
LCYVALDFEQEM ATAASSSSLEK	K7F239;K7F0D9;B3GS65; K7FID2;K7FVI4	2549. 1666	69.369
NYLAWYQQK	K7FYE6	1212. 5928	67.903
EDLLAEAWKED NSLESPR	K7FLD5	2100. 9964	65.408
EDITAITLRDTGC K	K7FU53;K7FU05	1591. 7876	59.645
SAETEVWYESIY QGAVFQLTK	K7FBM7	2448. 1849	58.508
DSMIWDCTCIGA GR	K7GDA6	1640. 6745	58.476
METMQELIPFAK EMLRQK	K7EZF8	2222. 1262	58.476
SESLVVCDVAED LVEK	K7FT14;K7FT26	1790. 8608	57.042
KVYPMECLIVDK	K7FY22	1493. 7622	53.028
DLKLENLMLDK DGHIK	K7FBR2;K7FKB4	1881. 0030	51.454

The identification score was obtained by searching the peptides through Andromeda, the search engine of the Maxquant software, and the score refers to the match between the theoretical and actual spectra.

Table S2. Predicted results of the bioinformatics analysis for the 47 peptides.

Number	Peptide sequence	Length	Toxicity	Solubility	Score[1]
1	DIASGLIGPLLICK	14	NO	Poor	0.7892
2	DSMIWDCTCIGAGR	14	YES	Poor	0.7228
**3**	**DDDIAALVVDNGSGMCK**	**17**	**NO**	**Good**	**0.6070**
4	AGTFFWSVVIPHER	14	NO	Poor	0.4946
5	DSTLIMQLLR	10	NO	Poor	0.4901
6	LIAELGSTSISNLGFR	16	NO	Poor	0.4410
**7**	**FDEILEASDGIMVAR**	**15**	NO	**Good**	**0.4260**
**8**	**IPAPDDPHE**	**9**	NO	**Good**	**0.4206**
**9**	**DLDVAILVGSMPR**	**13**	NO	**Good**	**0.4184**
**10**	**YEGWPEVVEMEGCIPQK**	**17**	NO	**Good**	**0.4062**
**11**	**GLCVDALIELSDENADWK**	**18**	NO	**Good**	**0.3921**
12	FSGSGSGTDFTLTISR	16	NO	Poor	0.3892
**13**	**FEVIEFDDGSGSVLR**	**15**	NO	**Good**	**0.3607**
**14**	**EDLLAEAWKEDNSLESPR**	**18**	NO	**Good**	**0.3165**
**15**	**LEYISEAAFEGLVNLR**	**16**	NO	**Good**	**0.3075**
**16**	**DICNDVLSLLEK**	**12**	NO	**Good**	**0.2904**
17	SQEPVTLDFLDAELENDIK	19	NO	Good	0.2706
18	EFEGEEEYLEILGITR	16	NO	Good	0.2628
19	NYLAWYQQK	9	NO	Poor	0.2523
20	METMQELIPFAKEMLRQK	18	NO	Good	0.2433
21	CLVGEFVSDVLLVPEK	16	NO	Good	0.2386
22	KVYPMECLIVDK	12	NO	Good	0.2330
23	EYVLPSFEVILEPSEK	16	NO	Good	0.2276
24	SYELPDGQVITIGNER	16	NO	Good	0.2062
25	DLKLENLMLDKDGHIK	16	NO	Good	0.1994
26	SESLVVCDVAEDLVEK	16	NO	Good	0.1856

Number	Peptide sequence	Length	Toxicity	Solubility	Score[1]
27	TYYIAAVEIEWDYSPNR	17	NO	Poor	0.1624
28	IMELQDILAK	10	NO	Good	0.1541
29	YTSALTYDGVLVMAETFR	18	NO	Poor	0.1541
30	EEEEVLDQGDFYSLISK	17	NO	Good	0.1499
31	ELENLAAMDLELQK	14	NO	Good	0.1491
32	EDITAITLRDTGCK	14	NO	Good	0.1483
33	YEELQLTAGR	10	NO	Good	0.1409
34	TIAVLLDDILQR	12	NO	Good	0.1277
35	FYPEDVSEELIQDITQR	17	NO	Good	0.1253
36	ADQLTEEQIAEFK	13	NO	Good	0.1219
37	REEEEVLDQGDFYSLISK	18	NO	Good	0.1170
38	DLADELALVDVIEDK	15	NO	Good	0.1072
39	EITVATGGFIYTGEVVHR	18	NO	Poor	0.1020
40	ATVHIQVNDVNEYAPVFK	18	NO	Poor	0.1011
41	LYTLVLTDPDAPSR	14	NO	Good	0.0984
42	IDTIAADESFTQVDIGDR	18	NO	Good	0.0978
43	TAFDEAIAELDTLSEESYK	19	NO	Good	0.0938
44	DLYANTVLSGGTTMYPGIADR	21	NO	Poor	0.0898
45	SAETEVWYESIYQGAVFQLTK	21	NO	Poor	0.0818
46	TITLEVEPSDTIENVK	16	NO	Good	0.0518
47	LCYVALDFEQEMATAASSSSLEK	23	NO	Good	0.0280

1.potential bioactive peptide score.

2.Peptides screened bioinformatcs for subsequent activity evaluation are marked with "black bold".
